# Understanding the dynamics driving obesity in socioeconomically deprived urban neighbourhoods: an expert-based systems map

**DOI:** 10.1186/s12916-024-03798-x

**Published:** 2025-01-07

**Authors:** Fleur ter Ellen, Joost Oude Groeniger, Karien Stronks, Luc L. Hagenaars, Carlijn B.M. Kamphuis, Joreintje D. Mackenbach, Mariëlle A. Beenackers, Karen Freijer, Pieter Coenen, Maartje Poelman, Karen M. Oude Hengel, Frank J. van Lenthe

**Affiliations:** 1 Department of Public Health, Erasmus MC University Medical Center, Rotterdam, the Netherlands; 2 Erasmus School of Social and Behavioural Sciences, Rotterdam, the Netherlands; 3 Department of Public and Occupational Health, Amsterdam UMC, Amsterdam, the Netherlands; 4 Department of Interdisciplinary Social Science, Utrecht University, Utrecht, the Netherlands; 5 Department of Epidemiology & Data Science, Amsterdam UMC, Amsterdam, the Netherlands; 6 Amsterdam Public Health Research Institute, Amsterdam, the Netherlands; 7 Partnerschap Overgewicht Nederland (PON), Erasmus MC University Medical Center, Rotterdam, the Netherlands; 8 Societal Participation and Health, Amsterdam Public Health Research Institute, Amsterdam, the Netherlands; 9 Chair Group Consumption and Healthy Lifestyles, Wageningen University & Research, Wageningen, the Netherlands; 10 Department of Work Health Technology, Netherlands Organisation for Applied Scientific Research TNO, Leiden, the Netherlands

**Keywords:** System dynamics, Complex system, Group Model Building, Obesity, Causal Loop Diagram, Neighbourhood environment, Socioeconomic inequalities

## Abstract

**Background:**

Over the past decades, the prevalence of obesity among adults has rapidly increased, particularly in socioeconomically deprived urban neighbourhoods. To better understand the complex mechanisms behind this trend, we created a system map exposing the underlying system driving obesity prevalence in socioeconomically deprived urban neighbourhoods over the last three decades in the Netherlands.

**Methods:**

We conducted Group Model Building (GMB) sessions with a group of thirteen interdisciplinary experts to develop a Causal Loop Diagram (CLD) of the obesogenic system. Using system-based analysis, the underlying system dynamics were interpreted.

**Results:**

The CLD demonstrates the food environment, physical activity environment, socioeconomic environment and socio-political environment, and their interactions. We identified the following overarching reinforcing dynamics in the obesogenic system in socioeconomically deprived urban neighbourhoods: (1) adverse socioeconomic conditions and an unhealthy food environment reinforced each other, (2) increased social distance between social groups and adverse socioeconomic conditions reinforced each other and (3) increased social distance between institutions and communities and the normalisation of unhealthy behaviours reinforced each other. These deeper system dynamics further reinforced chronic stress, sedentary behaviour, sleeping problems, unhealthy diets and reduced physical activity over time. In turn, these dynamics led to the emergent result of rising obesity prevalence in socioeconomically deprived urban neighbourhoods over the past decades.

**Conclusions:**

Our study sheds light on the system dynamics leading to neighbourhoods with an unhealthy food environment, challenging socioeconomic conditions, a widening distance between social groups and an infrastructure that discouraged physical activity while promoting sedentary behaviour. Our insights can form the basis for the development of an integrated approach aimed at reshaping the obesogenic system in socioeconomically deprived urban neighbourhoods.

**Supplementary Information:**

The online version contains supplementary material available at 10.1186/s12916-024-03798-x.

## Background

A global rise in the prevalence of obesity over recent decades has led to about 13% of the adult population living with obesity currently, resulting in substantial societal burden [1–3]. This rise in obesity prevalence is unevenly distributed across population subgroups, with higher obesity rates among people with a lower socioeconomic position and people living in more socioeconomically deprived neighbourhoods compared to people with a higher socioeconomic position and people living in more affluent neighbourhoods [1, 4–6]. For example, in the Netherlands 15% of adults aged 18 and older suffered from obesity in 2023, a threefold increase since the early 1980s, and those with a primary or lower secondary educational degree were twice as likely to have obesity than those was a tertiary educational degree [7]. To date, preventive interventions and policies have not been able to curb the rising obesity prevalence, nor to reduce its socioeconomic gradient [1, 8]. A possible explanation is that obesity prevention strategies often prioritise individual health behaviours, and thereby treating obesity as an individual problem rather than a societal responsibility [9, 10]. This approach neglects the impact of adverse trends in our socioeconomic, cultural, food and built environment, which have driven the significant increase in obesity rates. Factors such as the increased use of technology have reduced physical activity, while the practices of the commercial food industry have led to growing exposure to fast food restaurants and digital food marketing influenced unhealthy eating habits [11–16]. 

Over the past decades, the global food environment has changed significantly, with a notable rise in the production, supply, marketing and consumption of ultra-processed foods and beverages [17]. These ultra-processed foods, typically high in fat, salt and sugar, now constituting a substantial portion of the diet in high-income countries, have been linked to an increased risk of obesity and chronic diseases [18–20]. Additionally, 79% of supermarket products in the Netherlands do not contribute to a healthy diet [21]. Also the food landscape in the Netherlands has changed considerably. Between 2004 and 2018, there was an increase in delivery services, takeaway outlets and restaurants, while the number of local food providers declined [14]. Consequently, most Dutch people do not adhere to dietary guidelines, with adherence being particularly low among lower socioeconomic groups [22]. Studies indicate that the healthiness of food environments varies across neighbourhoods, with a higher concentration of supermarkets, takeaways and fast-food outlets in lower-income areas, both globally [1, 23] and in the Netherlands [14]. Furthermore, a growing body of evidence shows how our food environments shape social norms about dietary behaviour and body weight [24–28]. Prevailing social norms may influence unhealthy food choices and higher body weight in some socio-cultural environments [29, 30], while cultivating thinness and healthy food repertoires in others [31–33]. As such, food environments not only contribute to a global rise in obesity, but also perpetuate class distinctions and health inequalities [34].

Recent research has begun adopting a ‘systems lens’ to better understand the interconnectedness of underlying causes of obesity. Drawing from systems science, this work conceptualises the increasing rates of obesity as an undesirable outcome of a larger complex adaptive system [35–37]. Within this complex adaptive system, the various factors influencing obesity prevalence are interconnected and changes in one part can trigger intended or unintended consequences in other parts of the system over time [38]. The Foresight obesity map was among the first that applied a system science method to develop a holistic system map of interconnected drivers of obesity [39]. This map supports the view that many current interventions only target parts of the system that promote population level obesity, referred to as the obesogenic system [40]. Nevertheless, the Foresight obesity map has been subject to criticism due to its emphasis on individual energy imbalance [11, 41]. This map also neglects the variations in obesity prevalence across areas. Recent studies have applied systems mapping methods to illustrate and understand the complex system of childhood and adolescent obesity [42, 43], or how the mechanisms behind the food environment drive unhealthy dietary intake [13, 44, 45]. To date, a comprehensive understanding which dynamics drive the prevalence of adult obesity in deprived urban neighbourhoods is still lacking. This understanding is crucial because despite the efforts made by local authorities to improve the determinants of obesity in deprived urban neighbourhoods, these actions have not resulted in substantial reductions in obesity prevalence [1]. Bridging this knowledge gap will offer valuable insights for the development of effective policy strategies to address obesity in deprived urban neighbourhoods. The aim of this study was therefore to create a system map exposing how the dynamics within the underlying system have contributed to the rise in obesity prevalence in deprived urban neighbourhoods over the last three decades. To achieve this, our research question was formulated as follows: What are the dynamics that have been driving the rise in obesity prevalence in socioeconomically deprived urban neighbourhoods in the Netherlands over the past 30 years?

## Methods

We conducted Group Model Building (GMB), a participatory research method that combines systems science with group facilitation [46]. GMB allows participants to visualise their beliefs and assumptions about how a complex system works, also referred to as participants’ mental models [46, 47].

### Participants of the GMB sessions

We employed purposive sampling by intentionally inviting experts with diverse areas of knowledge, including health inequalities, overweight, the food environment, occupational health, physical exercise, the urban living environment and social determinants of health. We compiled a list of experts to invite, presenting the purpose of the GMB sessions within the invitation. Subsequently, some of these experts were recommended by colleagues to participate in the sessions. Over a span of 9 months, thirteen experts took part, spread over two plenary sessions and four subgroup sessions (see *Additional file 1: Information of experts*). The main goal of these sessions was to build a mutual understanding and integrate knowledge about the obesogenic system in socioeconomically deprived urban neighbourhoods [48].

### The process of GMB

Following GMB protocols, an iterative process of participatory sessions with experts and pre-meeting and follow-up activities by the core modelling group (FtE, JOG, KS, LH, KOH, FJvL) was conducted [46]. Each GMB session was designed based on scripts, in accordance with structured techniques to facilitate individual and group activities [49]. The scripts used from the online tool *Scriptapedia* were first divergent to collect broad ideas and interpretations of the causes and consequences of the problem, followed by convergent scripts to focus on narrowing down, resolving disagreement and reaching consensus [49]. After each GMB session, the core modelling group refined the systems map and sent workbooks to the experts, including the summary report of the GMB, considerations and discussion topics for the next meeting [46]. After the final session, the results were shared with the experts to ascertain their alignment with the interpretations made by the core modelling group. *Additional file 2:* *GMB procedure* presents the procedure in detail.

### GMB to develop the Causal Loop Diagram (CLD)

The experts qualitatively developed a conceptual model of the obesogenic system in deprived urban neighbourhoods in the form of a ‘Causal Loop Diagram’ (CLD). A CLD is a system dynamics tool aimed at contributing to an understanding and visualisation of how complex systems work [50]. Table 1 shows the process of the development of our CLD following a GMB procedure.

**Table 1:** Development of the Causal Loop Diagram (CLD) by following a Group Model Building (GMB) procedure

CLD development process [50, 51]	Corresponding GMB procedure [49, 52]
Defining the systems boundaries	Discussion within the core modelling group
Extracting factors and connections to conceptualise the CLD	The use of systems mapping tools during first GMB session: Graphs Over Time, Nominal Group Technique, Connection circle exercise
Refining the initial CLD and identifying the subsystems	Discussions within the core modelling group Second GMB session with experts: Model review exercise
Refining the subsystems and identifying crucial feedback loops within subsystems	Discussion within the core modelling group Separate GMB sessions with all subgroups: Causal Mapping in Small Groups exercise
Identifying and analysing key dynamics in the subsystems and whole system CLD	Systems-based analysis with the core modelling group

A CLD presents the factors, connections and feedback loops that explain how a system behaves over time [50]. The factors in a CLD are the elements that influence each other within the system and form cause and effect relationships. Connections are represented as arrows between factors, and these show *how* a change in one factor causes a change in another factor. In a CLD, these arrows present hypothesised causal relationships between the different factors [53]. Multiple connections can form feedback loops, which are cycles where the factors continuously affect each other. Feedback loops in CLDs are either balancing or reinforcing. Reinforcing loops amplify the system’s behaviour over time whereas balancing feedback loops maintain stability in the system by resisting changes [54].

### Defining systems boundaries

The core modelling team defined the boundaries of the system, referring to the geography, concepts and/or processes that are or are not part of the system [35]. We focussed on key determinants that (1) influenced or were influenced by the rise in obesity prevalence over the past 30 years and/or (2) factors relevant for socioeconomically deprived urban neighbourhoods. A socioeconomically deprived urban neighbourhood was defined as a neighbourhood with a high concentration of people with a low socioeconomic position [55, 56], whereby we focused on urban municipalities (medium and large-sized municipalities) in the Netherlands [57, 58]. To maintain readability, we will continue to use the term ‘deprived’ throughout the paper when referring to socioeconomically deprived urban neighbourhoods. Biological factors (e.g. hormone levels, brain chemistry, genetic influences) were out of scope.

### Conceptualising the CLD

During the first GMB session, the experts became familiar with a more systemic and dynamic way of thinking by individually drawing ‘graphs-over-time’ of the most important factors that have influenced or are influenced by the rise of obesity over the past 30 years [49]. Following that, the experts within their subgroups assigned priority to these factors and subsequently conveyed their most relevant factors to the entire group. Ultimately, they collectively identified interconnections among these factors [49].

### Identifying subsystems

After the first GMB session, the core modelling group observed that four subsystems appeared: the food environment, the physical activity environment, the socioeconomic environment and the socio-political environment. Based on their expertise, experts were divided into four subgroups during the second session and critically assessed the subsystems. At the end of the session, subgroups presented their findings plenary and gathered insights from other experts.

### Refining the subsystems and identifying feedback loops

At the end of the second GMB session, some experts indicated that a ‘fact-check’ was necessary for factors of which the change over time was doubtful. For these factors, the core modelling group checked Dutch databases (e.g. Statistics Netherlands), scientific literature and policy documents for validation. In the last GMB session, experts refined and agreed on central feedback processes in the subsystem and suggested links to other subsystems.

### Identifying and analysing key dynamics

In line with previous systems science work, we differentiated between the system’s structure (factors, connections and feedback loops) and its functioning (deeper dynamics and their meaning) during the analysis [43, 59]. The GMB sessions were conducted to conceptualise the *structure* of the subsystems: the factors, connections and feedback loops that were relevant to the experts. To gain further insights into the *functioning* of each subsystem, the core modelling group combined identified mechanisms in each subsystem, referring to segments of larger processes in the subsystem [60]. Subsequently, the four subsystems were merged into a whole system CLD using online system mapping software *Kumu* and by integrating expert input to adjust connections [61]. Finally, to extract the overarching dynamics for a deeper understanding of the system’s functioning, the core modelling group analysed and discussed: (1) how factors present in more than one subsystem influence the identified subsystem mechanisms, and (2) how the separate subsystem mechanisms feed into each other.

## Results

The underlying system contributing to the rise in obesity prevalence in deprived urban neighbourhoods shows the interconnected structure of 63 factors and four subsystems (*Additional file 3: Whole system CLD*). An interactive visualisation of the CLD can be found here: Obesogenic system in socioeconomically deprived urban neighbourhoods in the Netherlands. All key feedback loops and the factor definitions are provided in *Additional file 4: Feedback loops and mechanisms* and *Additional file 5: Definitions of factors*.

### The subsystem CLDs

#### Subsystem of the food environment

**Fig. 1:**
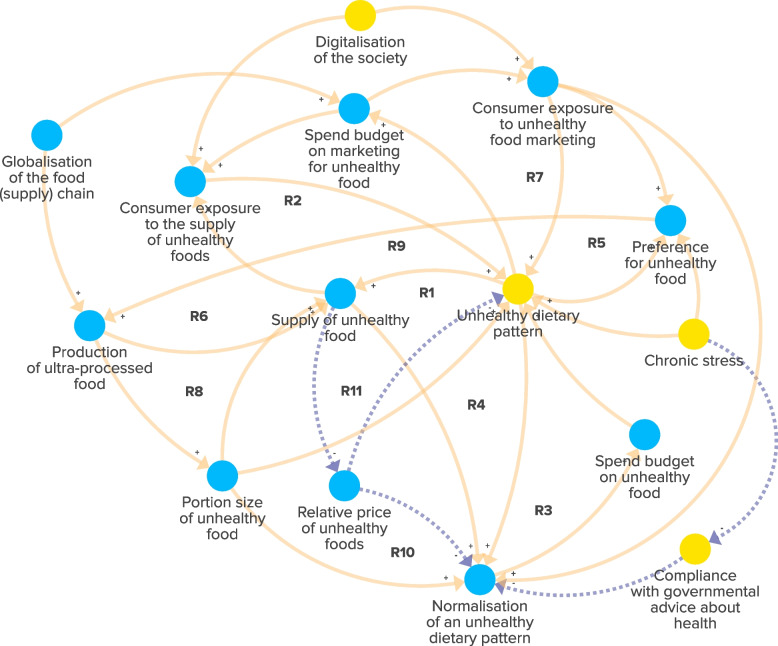
The subsystem of the food environment in deprived urban neighbourhoods in the Netherlands. Blue factors are unique to the subsystem. Yellow factors are ‘link elements’ that signal the presence of a factor in another subsystem. Dotted arrows: negative connections, indicating that the relationship between factors moves in the opposite direction. Solid arrows: positive connections, indicating that the relationship between factors moves in the same direction. ‘R1–R11’: reinforcing feedback loops within the subsystem

The experts emphasised the increased supply of unhealthy food in deprived urban neighbourhoods over the past decades. This trend is driven by the globalisation of the food chain and digitalisation of our society. Due to the profit-driven food industry and increased consumer exposure to unhealthy food with relative low prices, unhealthy dietary patterns have become increasingly prevalent. A higher prevalence of unhealthy dietary patterns in the neighbourhood in turn changed social norms around food consumption, which further fuelled the increasing supply-and-demand loop of unhealthy food (Fig. 1, R1-R4). The experts emphasised that while these trends are also seen in other contexts, their impact on obesity prevalence is more pronounced in deprived urban neighbourhoods due to interactions with deprived socioeconomic conditions of communities. Experts also discussed that the consumption of (larger portions of) unhealthy foods promoted a preference for high-calorie, sweet, salty and/or ultra-processed foods. This increased preference increased the demand for unhealthy food and further promoted the supply of these foods in the neighbourhood (Fig. 1, R5, R6, R9–R11). Additionally, increased exposure to online and offline marketing amplified unhealthy dietary patterns and preferences (Fig. 1, R7, R8). While governmental advice about healthy nutrition was provided, this advice was not able to compete with the negative effects that the marketing of and exposure to unhealthy foods had on social norms around food consumption. In sum, the interplay between supply-and-demand for unhealthy food within the food environment has created a reinforcing structure that has led to increasingly unhealthy dietary patterns, thereby promoting obesity in the deprived urban neighbourhoods.

#### Subsystem of the physical activity environment

**Fig. 2:**
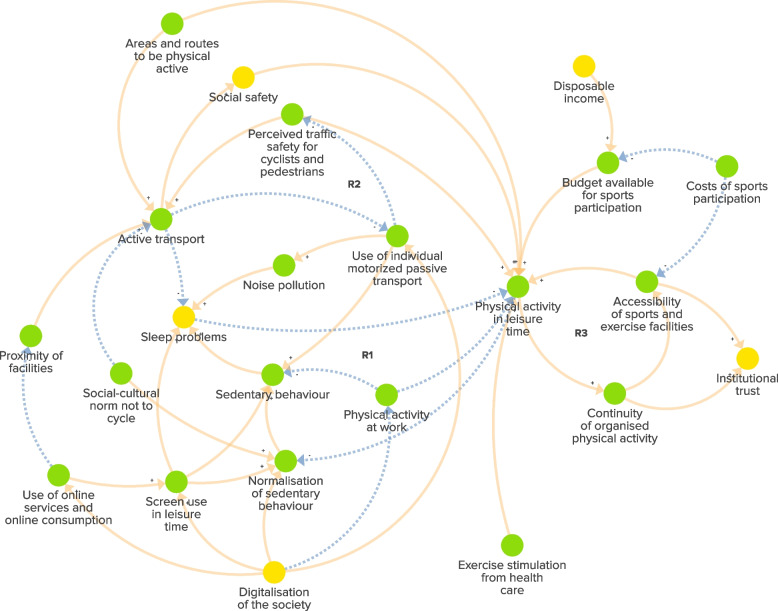
The subsystem of the physical activity environment in deprived urban neighbourhoods in the Netherlands. Green factors are unique to the subsystem. Yellow factors are ‘link elements’ that signal the presence of a factor in another subsystem. Dotted arrows: negative connections, indicating that the relationship between factors moves in the opposite direction. Solid arrows: positive connections, indicating that the relationship between factors moves in the same direction. ‘R1-R3’: reinforcing feedback loops within the subsystem

The experts pointed out that the ongoing digitalisation of society in recent decades has resulted in more screen time, increased use of online services and reduced physical activity, notably within workplace environments. As a result, this has caused changes in social norms within communities. Figure 2 demonstrates how this trend of normalised sedentary behaviour has negatively affected both sleep quality and in turn leisure-time physical activity (Fig. 2, R1). Furthermore, the experts discussed how the increased popularity of motorised vehicles such as scooters and motorcycles reduced active transport and undermined traffic and social safety in deprived urban neighbourhoods. The vicious cycle of how lower experienced traffic safety by cyclists and pedestrians reduced active transport, increased the use of individual motorised passive vehicles and lowered traffic safety even more (Fig. 2, R2). The experts noted that this hindered the impact of policy developments in public outdoor spaces, such as adding more parks and walking routes, to increase physical activity levels. Experts noted that leisure-time physical activity levels are notably lower in deprived urban neighbourhoods compared to more affluent neighbourhoods, leading to a diminished demand for exercise facilities. As a result, the continuity and accessibility of these facilities have remained limited or even declined. Additionally, many facilities have been moved to the outskirts of the city due to urban space constraints, often misaligning with community needs and fostering institutional distrust. Experts emphasised that the insufficient availability, accessibility and affordability of sports facilities provided by associations or gyms in many neighbourhoods contributed to low levels of physical activity, and reinforced the normalisation of sedentary behaviours (Fig. 2, R3). In summary, the dynamics within this subsystem have resulted in an infrastructure (comprising built environment and facilities) that has consistently maintained low levels of physical activity while promoting sedentary behaviour. This has contributed to the rise of obesity in deprived urban neighbourhoods over the past decades.

**Fig. 3:**
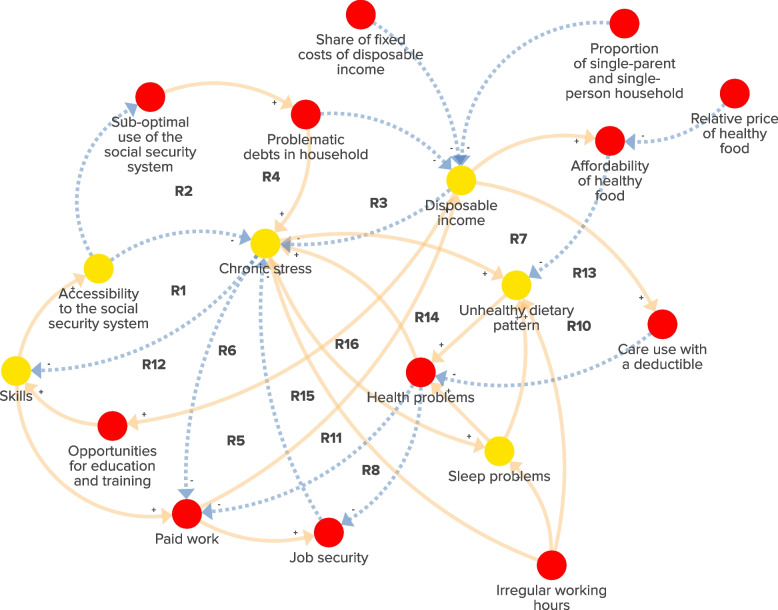
The subsystem of the socioeconomic environment in deprived urban neighbourhoods in the Netherlands. Red factors are unique to the subsystem. Yellow factors are ‘link elements’ that signal the presence of a factor in another subsystem. Dotted arrows: negative connections, indicating that the relationship between factors moves in the opposite direction. Solid arrows: positive connections, indicating that the relationship between factors moves in the same direction. ‘R1–R16’: reinforcing feedback loops within the subsystem

#### Subsystem of the socioeconomic environment

The experts emphasised several societal trends that negatively affected income security in deprived urban neighbourhoods over the past decades, such as an increased number of single-person and single-parent households, increased fixed household costs (e.g. rent, electricity) and an increase in jobs with temporary contract or multi-job holding. The experts also highlighted that the digitalisation of the Dutch social security system has decreased its accessibility, leading to sub-optimal use of government income support, especially for lower socioeconomic groups. Consequently, fewer residents received the benefits they were entitled to, increasing their risk of debt and chronic financial stress. This stress reduced individuals’ energy and motivation to navigate the complexities of the Dutch social security system, which further lowered their disposable income. Hence, chronic stress and low income created vicious cycles: stress hampered the ability to develop essential skills, including digital and financial literacy, while reduced disposable income limited opportunities for further education. This, in turn, worsened the difficulty of navigating the complex social security system, making it even less accessible for those in need. As a result, stress was further intensified, possibilities to obtain (new) paid work decreased and income growth continued to be constrained, sustaining the vicious cycles (Fig. 3, R1–R6, R12). The experts stressed the emergence of job insecurity (e.g. part-time work, temporary work and multi-jobbing). This led to an increase in chronic stress, which in turn impacted unhealthy dietary patterns and sleeping problems, promoting obesity (Fig. 3, R7–R8, R14). The experts emphasised that, even if employment was stable, irregular working hours also led to unhealthy eating habits, stress and sleep issues, further undermining good health and in turn further elevated the risk of obesity. Avoiding health care because of costs (including direct costs for health care access, but also travel costs or lost income by not being able to work) also led to increasing health problems, and subsequently negatively impacted chronic stress, job security and disposable income. These mechanisms further undermined pathways leading to higher disposable income (Fig. 3, R10, R15, R16). Increased chronic stress and reduced opportunities for education and training undermined people’s ability for skills development. These pathways directly and indirectly affected health, leading to a decline in employment and income security. To conclude, the dynamics in the socioeconomic environment led to an accumulation of chronic stress that contributed to the deterioration of socioeconomic conditions (e.g. income, debts, employment and education opportunities) in deprived urban neighbourhoods. This accumulation of chronic stress contributed to the rise of obesity in these neighbourhoods over time.

#### Subsystem of the socio-political environment

**Fig. 4:**
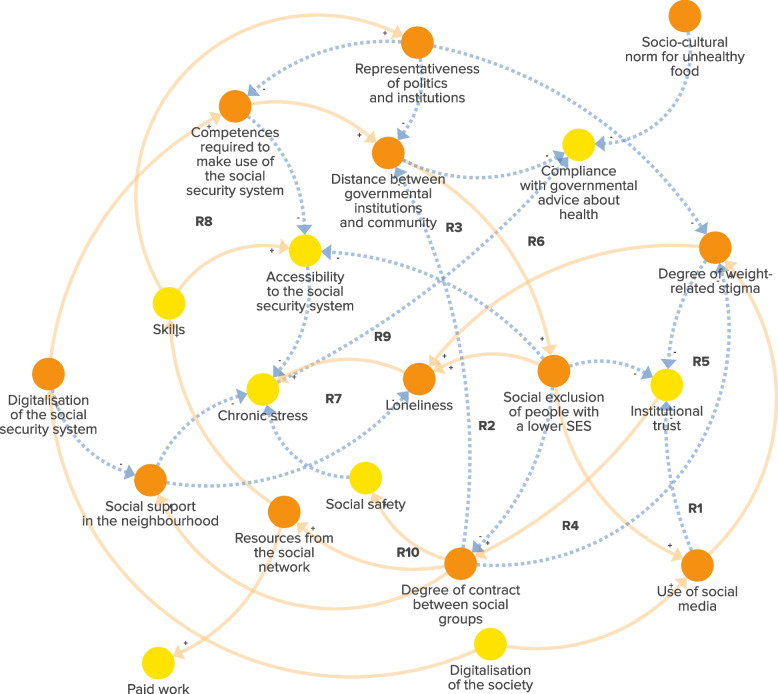
The subsystem of the socio-political environment in deprived urban neighbourhoods in the Netherlands. Orange factors are unique to the subsystem. Yellow factors are ‘link elements’ that signal the presence of a factor in another subsystem. Dotted arrows: negative connections, indicating that the relationship between factors moves in the opposite direction. Solid arrows: positive connections, indicating that the relationship between factors moves in the same direction. ‘R1–R10’: reinforcing feedback loops within the subsystem

The experts highlighted the increased distance between political and governmental institutions and communities in deprived areas over the past decades. This was primarily attributed to reduced political and institutional representation of these communities. As a consequence, the experts reported the mismatch between the heightened financial and digital competences required to navigate through the social security system and the actual level of these competences within these communities (Fig. 4). The heightened requirements consequently limited the accessibility, comprehensibility and usability of social security benefits, information and services. As a result, these increased demands exacerbated chronic stress and reinforced the divide between social groups. According to the experts, the absence of effective communication and understanding between those in positions of power and residents in deprived urban neighbourhoods led to a lack of awareness regarding the challenges faced by communities. This resulted in misaligned policies and interventions that contributed to various health problems in these areas, including obesity. The growing gap in preferences, perspectives and practices between those who work for and within governmental and political organisations and communities in deprived urban neighbourhoods has further reinforced the social exclusion of these communities. This exclusion has reduced contact between social groups, diminishing opportunities for interaction and understanding, and increasing institutional distrust (Fig. 4, R2-R3). This is illustrated by the reinforcing feedback loops where institutional distrust is further fuelled by increased social media use (predominantly interactions with like-minded individuals), similar to how weight-related stigma is fuelled by increased social media use. Both processes reduced the degree of contact between social groups and further increased the distance between political and governmental institutions and communities (Fig. 4, R1, R4–R5), and increased chronic stress via reduced social support and social safety. Reduced social network resources hindered access to crucial information and resources beyond one’s immediate social circle. This impeded the acquisition of knowledge and skills necessary for participation in political and governmental institutions, thereby reinforcing the underrepresentation of communities in these spheres through several reinforcing pathways (Fig. 4, R6–R10). As a result, the distance between governmental institutions and communities further increased. In sum, the dynamics within the socio-political subsystem revealed a social structure that pushed social groups apart over the past decades. This division has led to chronic stress and the implementation of ineffective health interventions, both of which have contributed to the rise of obesity in these neighbourhoods over time.

### Key dynamics in the obesogenic system as a whole

This section describes the three key dynamics of the whole system in deprived urban neighbourhoods in the Netherlands (visualised in *Additional files 6: Key dynamic 1, Additional file 7: Key dynamic 2* and *Additional file 8: Key dynamic 3).*

#### Dynamic 1: Adverse socioeconomic conditions and an unhealthy food environment reinforce each other.

The combination of reinforcing feedback loops demonstrates how the prevalence of obesity in deprived urban neighbourhoods increased due to interactions between supply-and-demand processes of unhealthy food, amplified chronic stress, and unhealthy dietary patterns. More specifically, limited budgets for healthy food, exposure to (the promotion of) unhealthy food, and chronic stress contributed to unhealthy dietary patterns (*Additional file 6: Key dynamic 1*). As unhealthy dietary patterns became more prevalent in deprived neighbourhoods, the preference and demand for unhealthy foods increased, exacerbating health problems in the community. This, in turn, further perpetuated adverse socioeconomic conditions, reinforcing the demand for unhealthy foods. The dynamics reveal that a high demand for unhealthy food resulted in an increased supply, which kept the prices of unhealthy food low and affordable, reinforcing unhealthy food consumption. The normalisation of an unhealthy diet contributed to worsened health problems, leading to reduced opportunities on the labour market, and consequently to a decrease in disposable income and an increase in chronic stress.

#### Dynamic 2: Increased social distance between social groups and adverse socioeconomic conditions reinforce each other

Reinforcing pathways show how declines in social resources, social support within and between groups, and unfavourable economic conditions were reinforced by each other, leading to increased chronic stress. Reduced contact *between* social groups limited access to resources related to work, education and other opportunities beyond those available *within* the social network of individuals living in deprived urban neighbourhoods. This lack of access to resources further hindered socioeconomic opportunities (*Additional file 7: Key dynamic 2*). Difficulties in acquiring necessary skills and accessing the social security system exacerbated debts, financial stress and limited socioeconomic opportunities. Fewer opportunities in the deprived urban neighbourhoods undermined possibilities for residents to end up in (high) positions in political institutions. The underrepresentation of communities in positions of power led to unintended outcomes, including misaligned policies, requirements and urban planning. These reinforcing processes widened the gap between communities and government institutions, leading to increased institutional distrust and distance between social groups. This worsened unfavourable socioeconomic conditions and chronic stress, increasing the risk of obesity in deprived urban neighbourhoods.

#### Dynamic 3: Increased social distance between institutions and communities and the normalisation of unhealthy behaviours reinforce each other

The CLD demonstrates how an increasing divide between political institutions and the lifeworld’s of communities resulted in inadequate responses to social problems and health challenges within the neighbourhood (*Additional file 8: Key dynamic 3*). This situation further contributed to political distrust and widened the gap between communities and the government. Additionally, the misalignment between governmental strategies aimed at improving population health and the preferences and practices of communities led to a further normalisation of unhealthy behavioural patterns. The risk of developing obesity increased because of these pathways that continued reinforcement of unhealthy behaviours in the neighbourhood.

## Discussion

### The functioning of the obesogenic system in deprived urban neighbourhoods

The four subsystem CLDs visualise dynamics that contributed to the rise of unhealthy dietary patterns and sedentary behaviour, low levels of physical activity, a widening gap between social groups and the accumulation of stressors that led to increased adverse socioeconomic conditions over the past 30 years. By conducting a systems-based analysis, we identified deeper system reinforcing dynamics between (1) adverse socioeconomic conditions and an unhealthy food environment, (2) increased social distance between social groups and adverse socioeconomic conditions and (3) increased social distance between institutions and communities and the normalisation of unhealthy behaviours. These dynamics further reinforced chronic stress, sedentary behaviour, sleeping problems, unhealthy diets and reduced physical activity, in turn, amplifying obesity in deprived urban neighbourhoods over the past decades.

#### The dynamic interplay between socioeconomic conditions and the neighbourhood food environment

In recent decades, global food supply has undergone changes, resulting in increased availability of cheaper unhealthy food [11, 36], also in deprived neighbourhoods in the Netherlands [14, 62]. The driving force behind this influx has been shown to be the pursuit of economic growth by transnational ultra-processed food companies. These companies promote products that yield high profits due to their hyper-palatability and long shelf-life, characteristics that also make their products contribute to causing obesity [63]. Notably, such strategies have intentionally disproportionately targeted communities in more deprived areas with limited resources [8]. As illustrated in our CLD, by capitalising on the socioeconomic vulnerabilities associated with living on a low income, the food industry and retail has perpetuated the reinforcing supply-and-demand process of unhealthy food in these neighbourhoods over the past decades. Moreover, the food and beverage industry’s marketing and pricing strategies have consequently played a significant role in shaping social norms related to dietary patterns and consumption. These trends are reflective of a prevailing societal paradigm that prioritises economic growth over public health [13, 64, 65].

#### The dynamic interplay between segregation, socioeconomic conditions and health behaviours

In the Netherlands, a shift towards a more individualistic political ideology has led to decreased government engagement in income, work and health, emphasising personal responsibility and the need for individual skills to navigate complex social and public services [66–68]. As a result, people became more reliant on the resources within collective social networks for enhancing health and work in deprived areas. The revealed dynamics in our CLD emphasise the significance of social capital—the collective benefits that arise from relationships, connections and social networks within a community—in shaping inequalities in obesity [69, 70]. Limited access to socioeconomic resources restricts individuals’ ability to effectively cope with stressors, leading to a reinforcement of chronic stress [71]. Chronic stress emerged as a result of various dynamics in our CLD and in turn feeds several processes that reinforce obesity, such as a preference for unhealthy food and sleep problems [72]. We acknowledge that by excluding biological systems in the CLD, we overlooked more detailed feedback processes demonstrating how an increase in chronic stress perpetuates obesity through physiological mechanisms [73]. The system dynamics also illustrate how reduced contact between social groups restricts access to resources and information needed to gain positions of power in politics and institutions [70]. This has had far-reaching consequences for policies developed by institutions dominated by tertiary educated individuals who have become socially and culturally distant from communities in deprived areas [74]. A lack of representation in government institutions further perpetuates policy decisions and health and social interventions that do not align with the needs and practices in the neighbourhood. This has resulted in a failure to adequately alleviate the underlying causes of chronic stress and other health problems in deprived neighbourhoods and fostered institutional distrust. In line with previous work, our dynamics demonstrate that institutional distrust could further decrease compliance with governmental health advice [75]. Although we deliberately chose to not include the emergent outcome of the system’s functioning (obesity prevalence) as a factor in the CLD, the dynamics highlight that a high obesity prevalence in deprived urban neighbourhoods also reinforces the system itself. Through its impact on social norms, stigmatisation, chronic stress and other health problems, socioeconomic inequalities in obesity will be amplified, creating a cycle that perpetuates this public health problem [3, 30, 76, 77].

### Implications

It is evident that a unilateral approach, such as focusing solely on the food industry, the physical environment or the socio-political situation, is insufficient to bring about systemic changes. To break the identified feedback loops, a comprehensive approach is necessary that considers the interaction between various systems underlying the rise of obesity prevalence in deprived urban neighbourhoods. For instance, tackling the demand for unhealthy food requires simultaneous efforts to improve socioeconomic situations as well as changes to the local food environment. Many reinforcing dynamics outlined in this paper manifest at a national or international scale, such as the accessibility of the social security system or macro-economic forces determining the production of ultra-processed foods. These forces interact with dynamics taking shape also at the local level, e.g. increased social distance between groups or the normalisation of unhealthy behaviours. This poses the question what local governments can or should do. The local level might not be the most appropriate level to address issues like the incentive structure of transnational food corporations. However, our results indicate the need for a joint strategy, in which activities tackling the influence of e.g. the ultra-processed food industry at the (supra)national level, go hand in hand with measures at the local level, including measures addressing socioeconomic conditions and norm-changing activities. Overall, this requires addressing the reign of free markets and individualism, and reverse the erosion of social policies within these communities [78]. Based on our identified dynamics, establishing trust between local authorities and communities, along with ensuring better representation of all groups in governmental organisations, is equally crucial.

To effectively address obesity, a comprehensive strategy must start with an inventory of current initiatives across various policy domains that influence the underlying dynamics. This assessment helps to identify existing gaps and informs targeted policies [79]. Emphasising collaboration between policy domains, an integrated approach should adopt the bidirectional relationship between health and other sectors, and public policy organisations and communities. Therefore, a ‘Health for All Policies’ approach [80] is needed in obesity prevention policy, which emphasises how obesity prevention contributes to other legitimate policy goals and solutions that are meaningful to affected communities [20]. Additionally, new initiatives should prioritise adaptability. Unlike static policies that struggle with unintended consequences, adaptive policies are designed to learn and evolve based on changing conditions within the system [81].

Our study was conducted in the context of the Netherlands, characterised by relatively small cities with closely located facilities and prevailing Dutch socio-cultural norms. The core mechanisms operating within and across the subsystems identified in our CLD may nevertheless also be relevant to deprived urban neighbourhoods in other high-income countries. The relative importance of these mechanisms may vary across different contexts however. Besides, other additional mechanisms not currently considered in our CLD could play a role in different contexts. For example, in the food environment, countries like the US experience more pronounced food deserts than the Netherlands due to the very different geographic and socioeconomic context [82, 83]. In terms of the physical activity environment, the Netherlands has a unique cycling culture supported by extensive infrastructure, which may differ from situations in other countries. Regarding the socioeconomic environment, the Netherlands benefits from a relatively generous social welfare system, including universal healthcare and various benefits, which may not be as prevalent elsewhere. Finally, the socio-political environment in the Netherlands is embedded in a parliamentary democracy marked by a pronounced influence of the highly educated, which might come with a different political engagement and representation than in other settings [84].

These contextual differences can affect the mechanisms within each subsystem and their interactions. Our findings offer a valuable foundation for exploring how specific contextual factors influence these dynamics. We recommend validating and incorporating context-specific mechanisms with input from national and local stakeholders before discussing potential intervention strategies. Engaging these stakeholders is crucial for effectively modifying system-reinforcing dynamics and ensuring that interventions are tailored to the local context and community’s actual experiences and needs [59, 79].

### Strengths and limitations

This study explored the rise of obesity by focusing on the dynamic interaction between upstream determinants of health by applying a system approach. This forced us to examine how determinants of obesity, including food and transportation systems, interact with broader socioeconomic and sociocultural determinants of health. Since the role of the private sector has been obscured in public health models with a traditional focus on individual lifestyle and social factors [85], we consider it a strength that we explicitly bridged the worlds of commercial and social determinants. A limitation of our CLD lies in its dependence on qualitative input from both the expert group and the core modelling group, without the inclusion of experiential knowledge from communities and policymakers. Consequently, the obesogenic system that emerged from the experts’ perspective might be vulnerable to ‘groupthink’ and the lack of diversity within the participant group [86]. Moreover, the 30-year period under consideration raises questions about whether all experts possessed sufficient knowledge to comprehensively assess this entire timeframe, potentially influencing the results. To enhance the robustness of our model, we actively addressed uncertainties through multiple sessions with the experts and reviewed trends and relationships in existing literature. However, it is important to note that validated effect sizes for factors and their relationships are missing in our CLD. While the construction of the CLD is grounded in expert research and practical experiences within relevant fields, it remains a reflection of the perspective held by a group of experts on the system in the Netherlands and should not be regarded as the absolute truth. Lastly, our study focused on the prevalence of obesity since it has been shown to be an important indicator of health problems at the population level. However, we acknowledge the extensive literature and ongoing scientific discussions that individuals with a high BMI do not always have poorer health outcomes [87–89].

## Conclusions

Our study sheds light on the complex dynamics through which increasingly unhealthy food environments, challenging socioeconomic conditions, widening distances between social groups and infrastructures that discourage physical activity and promote sedentary behaviour contributed in a mutually reinforcing manner to the rise of obesity in deprived urban neighbourhoods over the past three decades. Many of these dynamics are rooted on (supra)national levels but they interact and expose themselves at the neighbourhood level, reinforcing chronic stress, unhealthy dietary patterns, sedentary behaviour, sleeping problems and low physical activity levels. These insights can form the basis for the development of an integrated approach aimed at reshaping the functioning of the present obesogenic system in socioeconomically deprived urban neighbourhoods.

## Supplementary Information

Additional file 1: Information of experts.

Additional file 2: GMB procedure.

Additional file 3: Whole system CLD.

Additional file 4: Feedback loops and mechanisms.

Additional file 5: Definitions of factors.

Additional file 6: Key dynamic 1.

Additional file 7: Key dynamic 2.

Additional file 8: Key dynamic 3.

## Data Availability

No datasets were generated or analysed during the current study.

## Abbreviations

GMB: Group Model Building
CLD: Causal Loop Diagram

## References

[1] World Health Organization. WHO European Regional Obesity Report 2022. World Health Organization. Regional Office for Europe; 2022.

[2] OECD. The heavy burden of obesity: The economics of prevention, OECD health policy Studies, Paris: OECD Publishing; 2019. Available from: 10.1787/67450d67-en.

[3] Hecker J, Freijer K, Hiligsmann M, Evers SMAA. Burden of disease study of overweight and obesity; the societal impact in terms of cost-of-illness and health-related quality of life. BMC Public Health. 2022;22(1):46. 34996413 10.1186/s12889-021-12449-2 PMC8740868

[4] van Lenthe FJ, Mackenbach JP. Neighbourhood deprivation and overweight: the GLOBE study. Int J Obes Relat Metab Disord. 2002;26(2):234–40. 11850756 10.1038/sj.ijo.0801841

[5] Robertson A. Obesity and socio-economic groups in Europe: Evidence review and implications for action. 2007. Available from: http://ec.europa.eu/health/ph_determinants/life_style/nutrition/documents/ev20081028_rep_en.pdf.

[6] Cubbin C, Sundquist K, Ahlén H, Johansson SE, Winkleby MA, Sundquist J. Neighborhood deprivation and cardiovascular disease risk factors: protective and harmful effects. Scand J Public Health. 2006;34(3):228–37. 16754580 10.1080/14034940500327935

[7] Overgewicht Volwassenen: Informatie over Volksgezondheid en Zorg. [Adult Overweight: Information on Public Health and Care]; 2023. Available from: https://www.vzinfo.nl/overgewicht/volwassenen.

[8] Roberto CA, Swinburn B, Hawkes C, Huang TTK, Costa SA, Ashe M, et al. Patchy progress on obesity prevention: emerging examples, entrenched barriers, and new thinking. The Lancet. 2015;385(9985):2400–9. 10.1016/S0140-6736(14)61744-X 25703111

[9] Williams O, Fullagar S. Lifestyle drift and the phenomenon of “citizen shift” in contemporary UK health policy. Sociol Health Illn. 2019;41(1):20–35. 30073681 10.1111/1467-9566.12783

[10] Johnston L, Matteson C, Finegood D. Systems Science and Obesity Policy: A Novel Framework for Analyzing and Rethinking Population-Level Planning. Am J Public Health. 2014;104(7):1270–8. 24832406 10.2105/AJPH.2014.301884 PMC4056198

[11] Swinburn BA, Kraak VI, Allender S, Atkins VJ, Baker PI, Bogard JR, et al. The Global Syndemic of Obesity, Undernutrition, and Climate Change: The Lancet Commission report. The Lancet. 2019;393(10173):791–846. 10.1016/S0140-6736(18)32822-8 30700377

[12] Anderson Steeves E, Martins PA, Gittelsohn J. Changing the Food Environment for Obesity Prevention: Key Gaps and Future Directions. Curr Obes Rep. 2014;3(4):451–8. 25574452 10.1007/s13679-014-0120-0 PMC4283210

[13] Sawyer ADM, van Lenthe F, Kamphuis CBM, Terragni L, Roos G, Poelman MP, et al. Dynamics of the complex food environment underlying dietary intake in low-income groups: a systems map of associations extracted from a systematic umbrella literature review. Int J Behav Nutr Phys Act. 2021;18(1):96. 34256794 10.1186/s12966-021-01164-1 PMC8276221

[14] Pinho MGM, Mackenbach JD, den Braver NR, Beulens JJW, Brug J, Lakerveld J. Recent changes in the Dutch foodscape: socioeconomic and urban-rural differences. Int J Behav Nutr Phys Act. 2020;17(1):43. 32197651 10.1186/s12966-020-00944-5 PMC7083034

[15] World Health O. Digital food environments: factsheet. Regional Office for Europe: World Health Organization; 2021.

[16] Wood B, Robinson E, Baker P, Paraje G, Mialon M, van Tulleken C, Sacks G. What is the purpose of ultra-processed food? An exploratory analysis of the financialisation of ultra-processed food corporations and implications for public health. Glob Health. 2023;19(1):85. 10.1186/s12992-023-00990-1 PMC10644600 37957671

[17] Baker P, Machado P, Santos T, Sievert K, Backholer K, Hadjikakou M, et al. Ultra-processed foods and the nutrition transition: Global, regional and national trends, food systems transformations and political economy drivers. Obes Rev. 2020;21(12):e13126. 32761763 10.1111/obr.13126

[18] Monteiro CA, Cannon G, Lawrence M, Costa Louzada M, Pereira Machado P. Ultra-processed foods, diet quality, and health using the NOVA classification system. Rome: FAO; 2019. Available from: https://openknowledge.fao.org/server/api/core/bitstreams/5277b379-0acb-4d97-a6a3-602774104629/content.

[19] Touvier M, da Costa Louzada ML, Mozaffarian D, Baker P, Juul F, Srour B. Ultra-processed foods and cardiometabolic health: public health policies to reduce consumption cannot wait. BMJ. 2023;383:e075294. 10.1136/bmj-2023-075294 PMC10561017 37813465

[20] Hagenaars LL, Schmidt LA, Groeniger JO, Bekker MPM, Ter Ellen F, de Leeuw E, et al. Why we struggle to make progress in obesity prevention and how we might overcome policy inertia: Lessons from the complexity and political sciences. Obes Rev. 2024;25(5):e13705. 38424004 10.1111/obr.13705

[21] Poelman MP, Dijkstra SC, Djojosoeparto SK, de Vet E, Seidell JC, Kamphuis CBM. Monitoring van de mate van gezondheid van het aanbod en de promoties van supermarkten en out-of-home-ketens: Inzicht in de huidige stand van zaken en aanbevelingen voor het opzetten van een landelijke monitor [Monitoring the healthiness of offerings and promotions in supermarkets and out-of-home chains: insights into the current situation and recommendations for establishing a national monitoring system]: Wageningen University & Research; 2021. Available from: 10.18174/555613.

[22] Schuurman RWC, Beukers MH, Van Rossum CTM. Eet en drinkt Nederland volgens de Richtlijnen Schijf van Vijf?: Resultaten van de voedselconsumptiepeiling 2012–2016. [Do the Dutch eat and drink according to the Wheel of Five? Results of the Dutch National Food consumption Survey 2012-2016]. 2020. Available from: 10.21945/RIVM-2020-0082.

[23] Black C, Moon G, Baird J. Dietary inequalities: what is the evidence for the effect of the neighbourhood food environment? Health Place. 2014;27:229–42. 24200470 10.1016/j.healthplace.2013.09.015 PMC4948665

[24] Higgs S. Social norms and their influence on eating behaviours. Appetite. 2015;86:38–44. 25451578 10.1016/j.appet.2014.10.021

[25] Robinson E, Thomas J, Aveyard P, Higgs S. What everyone else is eating: a systematic review and meta-analysis of the effect of informational eating norms on eating behavior. J Acad Nutr Diet. 2014;114(3):414–29. 24388484 10.1016/j.jand.2013.11.009

[26] Prinsen S, de Ridder DTD, de Vet E. Eating by example. Effects of environmental cues on dietary decisions. Appetite. 2013;70:1–5. 23791633 10.1016/j.appet.2013.05.023

[27] De Ridder D, De Vet E, Stok M, Adriaanse M, De Wit J. Obesity, overconsumption and self-regulation failure: The unsung role of eating appropriateness standards. Health Psychol Rev. 2013;7(2):146–65.

[28] Van Rongen S, Poelman MP, Thornton L, Abbott G, Lu M, Kamphuis CBM, et al. Neighbourhood fast food exposure and consumption: the mediating role of neighbourhood social norms. Int J Behav Nutr Phys Act. 2020;17:1–9. 32404102 10.1186/s12966-020-00969-w PMC7218623

[29] Bahr DB, Browning RC, Wyatt HR, Hill JO. Exploiting social networks to mitigate the obesity epidemic. Obesity. 2009;17(4):723–8. 19148124 10.1038/oby.2008.615

[30] Crielaard L, Dutta P, Quax R, Nicolaou M, Merabet N, Stronks K, Sloot PMA. Social norms and obesity prevalence: From cohort to system dynamics models. Obes Rev. 2020;21(9):e13044. 32400030 10.1111/obr.13044 PMC7507199

[31] Oude Groeniger J, de Koster W, van der Waal J, Mackenbach JP, Kamphuis CBM, van Lenthe FJ. How does cultural capital keep you thin? Exploring unique aspects of cultural class that link social advantage to lower body mass index. Sociol Health Illn. 2020;42(7):1497–515. 32538479 10.1111/1467-9566.13120 PMC7586794

[32] Christensen VT, Carpiano RM. Social class differences in BMI among Danish women: applying Cockerham’s health lifestyles approach and Bourdieu’s theory of lifestyle. Soc Sci Med. 2014;112:12–21. 24788112 10.1016/j.socscimed.2014.04.017

[33] Schreiber K, Hausenblas HA. The truth about exercise addiction: Understanding the dark side of thinspiration. Lanham (MD): Rowman & Littlefield; 2015.

[34] Mollborn S, Pace JA, Rigles B. Children’s Health Lifestyles and the Perpetuation of Inequalities. J Health Soc Behav. 2024;0(0). 10.1177/00221465241255946 PMC11869505 40013477

[35] Lee BY, Bartsch SM, Mui Y, Haidari LA, Spiker ML, Gittelsohn J. A systems approach to obesity. Nutr Rev. 2017;75(suppl_1):94–106. 28049754 10.1093/nutrit/nuw049 PMC5207008

[36] Swinburn BA, Sacks G, Hall KD, McPherson K, Finegood DT, Moodie ML, Gortmaker SL. The global obesity pandemic: shaped by global drivers and local environments. The Lancet. 2011;378(9793):804–14. 10.1016/S0140-6736(11)60813-1 21872749

[37] Rutter H, Savona N, Glonti K, Bibby J, Cummins S, Finegood DT, et al. The need for a complex systems model of evidence for public health. Lancet. 2017;390(10112):2602–4. 28622953 10.1016/S0140-6736(17)31267-9

[38] Meadows DH. Thinking in systems- a primer. London: Earthscan; 2008.

[39] Vandenbroeck I, Goossens J, Clemens M. Foresight tackling obesities: future choices—obesity system atlas. Foresight Study, 2007. 10.1111/j.1467-789X.2007.00344.x 17316292

[40] Swinburn B, Egger G. Preventive strategies against weight gain and obesity. Obes Rev. 2002;3(4):289–301. 12458974 10.1046/j.1467-789x.2002.00082.x

[41] McGlashan J, Hayward J, Brown A, Owen B, Millar L, Johnstone M, et al. Comparing complex perspectives on obesity drivers: action-driven communities and evidence-oriented experts. Obes Sci Pract. 2018;4(6):575–81. 30574350 10.1002/osp4.306 PMC6298210

[42] Allender S, Owen B, Kuhlberg J, Lowe J, Nagorcka-Smith P, Whelan J, Bell C. A Community Based Systems Diagram of Obesity Causes. PLoS ONE. 2015;10(7):e0129683. 26153893 10.1371/journal.pone.0129683 PMC4496094

[43] Luna Pinzon A, Stronks K, Emke H, van den Eynde E, Altenburg T, Dijkstra SC, et al. Understanding the system dynamics of obesity-related behaviours in 10-to 14-year-old adolescents in Amsterdam from a multi-actor perspective. Front Public Health. 2023;11:1696. 10.3389/fpubh.2023.1128316 PMC10248031 37304107

[44] Friel S, Pescud M, Malbon E, Lee A, Carter R, Greenfield J, et al. Using systems science to understand the determinants of inequities in healthy eating. PloS one. 2017;12(11):e0188872-e. 29190662 10.1371/journal.pone.0188872 PMC5708780

[45] Wopereis TM, Dijkstra C, Wierda JJ, Rongen FC, Poelman MP. Systems thinking for local food environments: a participatory approach identifying leverage points and actions for healthy and sustainable transformations. Health Res Policy Syst. 2024;22(1):101. 39135050 10.1186/s12961-024-01199-3 PMC11318250

[46] Vennix JAM. Group model building: Facilitating team learning using system dynamics. Chichester; 1996.

[47] Ford DN, Sterman JD. Expert knowledge elicitation to improve formal and mental models. Syst Dynamics Rev: Jo Syst Dynamics Soc. 1998;14(4):309–40.

[48] Voinov A, Bousquet F. Modelling with stakeholders. Environ Model Softw. 2010;25(11):1268–81.

[49] Hovmand PS, Rouwette EAJA, Andersen DF, Richardson GP. Scriptapedia: Wikibooks; 2015. Available from: https://en.wikibooks.org/wiki/Scriptapedia.

[50] Barbrook-Johnson P, Penn AS. Systems Mapping: How to build and use causal models of systems. Springer Nature; 2022. Available from: 10.1007/978-3-031-01919-7.

[51] Crielaard L, Uleman JF, Châtel BDL, Epskamp S, Sloot PMA, Quax R. Refining the causal loop diagram: A tutorial for maximizing the contribution of domain expertise in computational system dynamics modeling. Psychol Methods. 2024;29(1):169–201. 10.1037/met0000484 35549316

[52] Hovmand PS. Group model building and community-based system dynamics process. Community based system dynamics: Springer; 2014. p. 17–30.

[53] Uleman JF, Stronks K, Rutter H, Arah OA, Rod NH. Mapping complex public health problems with causal loop diagrams. Int J Epidemiol. 2024;53(4):dyae091. 10.1093/ije/dyae091 PMC13377455 38990180

[54] Sterman JD. Systems Thinking and modeling for a complex world. McGraw-Hill Higher Education; 2000.

[55] Bak CK, Tanggaard Andersen P, Bacher I, Draghiciu BD. The association between socio-demographic characteristics and perceived stress among residents in a deprived neighbourhood in Denmark. Eur J Public Health. 2012;22(6):787–92. 22315461 10.1093/eurpub/cks004

[56] Arts K, van Gaalen R, van der Laan J, Linder F, Mol J, van Rooijen J, et al. Berekenwijze Sociaal Economische Status scores. [Calculation Method for SES Score]. The Hague/Heerlen: Statistics Netherlands; 2021. Available from: https://www.cbs.nl/nl-nl/maatwerk/2021/45/berekenwijze-ses-score-per-wijk-buurt.

[57] Statistics Netherlands (CBS). Stedelijkheid van een gebied. [Degree of urbanisation]. 2022. Available from: https://www.cbs.nl/nl-nl/onze-diensten/methoden/begrippen/stedelijkheid--van-een-gebied.

[58] Statistics Netherlands (CBS). 42 grootste steden. [42 largest cities]. 2017. Available from: https://www.cbs.nl/nl-nl/nieuws/2017/45/partners-steeds-vaker-beiden-hoogopgeleid/42-grootste-steden.

[59] Foster-Fishman PG, Nowell B, Yang H. Putting the system back into systems change: A framework for understanding and changing organizational and community systems. Am J Community Psychol. 2007;39(3–4):197–215. 17510791 10.1007/s10464-007-9109-0

[60] Waterlander WE, Singh A, Altenburg T, Dijkstra C, Luna Pinzon A, Anselma M, et al. Understanding obesity-related behaviors in youth from a systems dynamics perspective: The use of causal loop diagrams. Obes Rev. 2020;22(7):1–16. 10.1111/obr.13185 PMC8243923 33369045

[61] Kumu. 2022. Available from: https://kumu.io/.

[62] Molenberg F, Beenackers M, Mackenbach JD, Burdorf L, van Lenthe F. Is Rotterdam een fastfoodparadijs? De voedselomgeving van 2004 tot 2018. Rotterdam. [Is Rotterdam a fast food paradise?: The food environment between 2004 and 2018]. 2019. Available from https://cephir.nl/wp-content/uploads/Rotterdam-voedselomgeving_Rapport_CEPHIR-s.pdf.

[63] Gilmore AB, Fabbri A, Baum F, Bertscher A, Bondy K, Chang H-J, et al. Defining and conceptualising the commercial determinants of health. Lancet. 2023;401(10383):1194–213. 36966782 10.1016/S0140-6736(23)00013-2

[64] Rushton S, Williams OD. Frames, paradigms and power: global health policy-making under neoliberalism. Glob Soc. 2012;26(2):147–67.

[65] Kim J, de Leeuw E, Harris-Roxas B, Sainsbury P. Four urban health paradigms: the search for coherence. Cities. 2022;128:103806.

[66] Ridder Jd, Lvt H, Avd B. Burgerperspectieven. 2023;I(1):2023.

[67] Bussemaker J, ’S Jongers T, Vonk R. Gezondheidsverschillen voorbij. [Beyond health inequalities]. TSG Tijdschr Gezondheidswet. 2021;99:36–9. Available from: 10.1007/s12508-020-00291-7.

[68] Bovens M, Keizer A-G, Tiemeijer W. Weten is nog geen doen: een realistisch perspectief op redzaamheid. [Why knowing what to do is not enough: a realistic perspective on self-reliance]. Netherlands Scientific Council for Government Policy. The Hague; 2017. Available from: https://www.wrr.nl/publicaties/rapporten/2017/04/24/weten-is-nog-geen-doen.

[69] Carpiano RM. Toward a neighborhood resource-based theory of social capital for health: can Bourdieu and sociology help? Soc Sci Med. 2006;62(1):165–75. 15992978 10.1016/j.socscimed.2005.05.020

[70] McCartney G, Dickie E, Escobar O, Collins C. Health inequalities, fundamental causes and power: towards the practice of good theory. Sociol Health Illn. 2021;43(1):20–39. 33222244 10.1111/1467-9566.13181 PMC7894306

[71] Crielaard L, Nicolaou M, Sawyer A, Quax R, Stronks K. Understanding the impact of exposure to adverse socioeconomic conditions on chronic stress from a complexity science perspective. BMC Med. 2021;19(1):1–20. 34635083 10.1186/s12916-021-02106-1 PMC8507143

[72] Tamashiro KL, Sakai RR, Shively CA, Karatsoreos IN, Reagan LP. Chronic stress, metabolism, and metabolic syndrome. Stress. 2011;14(5):468–74. 21848434 10.3109/10253890.2011.606341

[73] van der Valk ES, van den Akker ELT, Savas M, Kleinendorst L, Visser JA, Van Haelst MM, et al. A comprehensive diagnostic approach to detect underlying causes of obesity in adults. Obes Rev. 2019;20(6):795–804. 30821060 10.1111/obr.12836 PMC6850662

[74] Bovens M, Wille A. Diplomademocratie: Over de spanning tussen meritocratie en democratie. [Diploma Democracy: On the tensions between meritocracy and democracy]. Prometheus; 2011

[75] van Meurs T, Oude Groeniger J, de Koster W, van der Waal J. An incongruous intervention: Exploring the role of anti-institutionalism in less-educated individual’s limited uptake of nutrition information. Sociol Health Illn. 2022;44(2):432–50. 35041765 10.1111/1467-9566.13430 PMC9303756

[76] Tomiyama AJ. Stress and Obesity. Annu Rev Psychol. 2019;70:703–18. 29927688 10.1146/annurev-psych-010418-102936

[77] Tomiyama AJ, Carr D, Granberg EM, Major B, Robinson E, Sutin AR, Brewis A. How and why weight stigma drives the obesity “epidemic” and harms health. BMC Med. 2018;16(1):123. 30107800 10.1186/s12916-018-1116-5 PMC6092785

[78] Oreskes N, Conway EM. The big myth: How American business taught us to loathe government and love the free market. USA: Bloomsbury Publishing; 2023.

[79] Nobles JD, Radley D, Mytton OT, team WSOp. The Action Scales Model: a conceptual tool to identify key points for action within complex adaptive systems. Perspect Public Health. 2021:17579139211006747. 10.1177/17579139211006747 PMC9720704 33998333

[80] Greer SL, Falkenbach M, Siciliani L, McKee M, Wismar M, Figueras J. From health in all policies to health for all policies. Lancet Public Health. 2022;7(8):e718–20. 35907422 10.1016/S2468-2667(22)00155-4 PMC9330081

[81] Carey G, Crammond B, Malbon E, Carey N. Adaptive policies for reducing inequalities in the social determinants of health. Int J Health Policy Manag. 2015;4(11):763. 26673337 10.15171/ijhpm.2015.170 PMC4629702

[82] Cummins S, Macintyre S. Food environments and obesity—neighbourhood or nation? Int J Epidemiol. 2006;35(1):100–4. 16338945 10.1093/ije/dyi276

[83] Helbich M, Schadenberg B, Hagenauer J, Poelman M. Food deserts? Healthy food access in Amsterdam. Appl Geogr. 2017;83:1–12.

[84] Bovens MAP. De diplomademocratie: over de spanning tussen meritocratie en democratie. [Diploma democracy: on the tensions between meritocracy and democracy]. B en M Tijdschr Beleid Politiek Maatschapp. 2006;33(4):205–18. Available from: https://dspace.library.uu.nl/handle/1874/19863.

[85] Maani N, Collin J, Friel S, Gilmore AB, McCambridge J, Robertson L, Petticrew MP. Bringing the commercial determinants of health out of the shadows: a review of how the commercial determinants are represented in conceptual frameworks. Eur J Pub Health. 2020;30(4):660–4. 31953933 10.1093/eurpub/ckz197 PMC7445044

[86] Lunenburg FC. Group decision making: The potential for groupthink. Int J Manag Business Admin. 2010;13(1):1–6.

[87] Putra ICS, Kamarullah W, Prameswari HS, Pramudyo M, Iqbal M, Achmad C, et al. Metabolically unhealthy phenotype in normal weight population and risk of mortality and major adverse cardiac events: A meta-analysis of 41 prospective cohort studies. Diabetes Metab Syndr. 2022;16(10):102635. 36240685 10.1016/j.dsx.2022.102635

[88] Fan J, Song Y, Chen Y, Hui R, Zhang W. Combined effect of obesity and cardio-metabolic abnormality on the risk of cardiovascular disease: a meta-analysis of prospective cohort studies. Int J Cardiol. 2013;168(5):4761–8. 23972953 10.1016/j.ijcard.2013.07.230

[89] Welk GJ, Blair SN. Physical Activity Protects against the Health Risks of Obesity. President's Council on Physical Fitness and Sports Research Digest. 2000;3(12).

