# Supplementary material for: Understanding the dynamics driving obesity in socioeconomically deprived urban neighbourhoods: an expert-based systems map
Source: BMC Med. 2025 Jan 7;23:2. doi: 10.1186/s12916-024-03798-x (PMC11705861; doi:10.1186/s12916-024-03798-x)
Supplement: Supplementary file 1 — Additional file 1: Information of experts. [file 12916_2024_3798_MOESM1_ESM.pdf]

Additional file 1: Information of experts involved in the project

Name	Organisation	Expertise
Carlijn Kamphuis	Utrecht University, Department of Interdisciplinary Social Science	Socioeconomic inequalities in health, the role of context and environmental factors for health and health-related behaviours, systems thinking.
Joreintje Mackenbach	Amsterdam UMC, Department of Epidemiology & Data Science	Epidemiology, prevention, food environments
Karen Hosper	Pharos Dutch Centre of Expertise on Health Disparities	Prevention, access to healthcare, health inequities, person-centred care, health literacy
Liesbeth Preller	Knowledge Centre for Sport & Physical Activity	Exercise behaviours
Mariëlle Beenackers	Erasmus MC, Department of Public Health	Urban health, healthy living environments, health inequalities
Pieter Coenen	Amsterdam UMC, Department of Public and Occupational Health; Societal Participation and Health, Amsterdam Public Health Research Institute	Occupational health, lifestyle
Karen Freijer	Partnership Overweight Netherlands (PON)	Overweight and obesity
Maartje Poelman	Chair Group consumption and Healthy Lifestyles, Wageningen University & Research	Food environments, prevention
Gerdine Fransen-Kuppens	Radboud university medical center, Academic Workplace Public Health AMPHI; Manager Municipality Health Service	Practice based research on public health, learning networks and monitoring of local healthy weight approaches
Joost Oude Groeniger	Erasmus MC, Department of Public Health; Erasmus University Rotterdam, Department of Public Administration and Sociology	Health inequalities, prevention, social determinants of health
Frank van Lenthe	Erasmus MC, Department of Public Health	Health inequalities, living environment, prevention, social determinants of health
Karien Stronks	Amsterdam UMC, Department of Public and Occupational Health	Health inequalities, systems thinking in public health, prevention
Karen Oude Hengel	Netherlands Organisation for Applied Scientific Research TNO Erasmus MC, Department of Public Health	Occupational health, lifestyle, health inequalities
