# Supplementary material for: Understanding the dynamics driving obesity in socioeconomically deprived urban neighbourhoods: an expert-based systems map
Source: BMC Med. 2025 Jan 7;23:2. doi: 10.1186/s12916-024-03798-x (PMC11705861; doi:10.1186/s12916-024-03798-x)
Supplement: Supplementary file 2 — Additional file 2: GMB procedure. [file 12916_2024_3798_MOESM2_ESM.pdf]

Additional file 2: GMB procedure to create the Causal Loop Diagrams

GMB session	Scripts used	Nature of group task	Use of script in GMB	Facilitating team
Session 1	Graphs over time	Divergent	Participants drew time graphs of the factors that have influenced or are influenced by the rise of obesity over the past 30 years to ensure a more systemic and dynamic way of thinking.	FtE, LH, LC
	Nominal Group Technique	Convergent	Participants prioritised factors first individually and thereafter in subgroups. The most relevant factors per subgroup were collected plenary and shown on the board for everyone to see.	
	Connection circle	Divergent	Participants identified important connections between factors plenary with the help of the facilitator and modeler.	
Session 2	Model Review	Convergent	Each subgroup reported their results back to the plenary group using flip charts and prepared worksheets.	
Session 2 & session 3	Causal Mapping in Small Groups	Divergent & convergent	Development and interpretation of subsystems during subgroup sessions.	FtE, LC, AL, RH

Scripts: structured techniques to facilitate individual and group activities during Group Model Building.

Divergent activities: to collect broad ideas and interpretations of the causes and consequences of the problem.

Convergent activities: to narrow down ideas, resolving disagreement and reaching consensus.

Reference Group Model Building scripts:

Hovmand PS, Rouwette EAJA, Andersen DF, Richardson GP, Kraus A (2013) Scriptapedia 4.0.6.
