# Supplementary material for: Understanding the dynamics driving obesity in socioeconomically deprived urban neighbourhoods: an expert-based systems map
Source: BMC Med. 2025 Jan 7;23:2. doi: 10.1186/s12916-024-03798-x (PMC11705861; doi:10.1186/s12916-024-03798-x)
Supplement: Supplementary file 3 — Additional file 3: Whole system CLD. [file 12916_2024_3798_MOESM3_ESM.pdf]

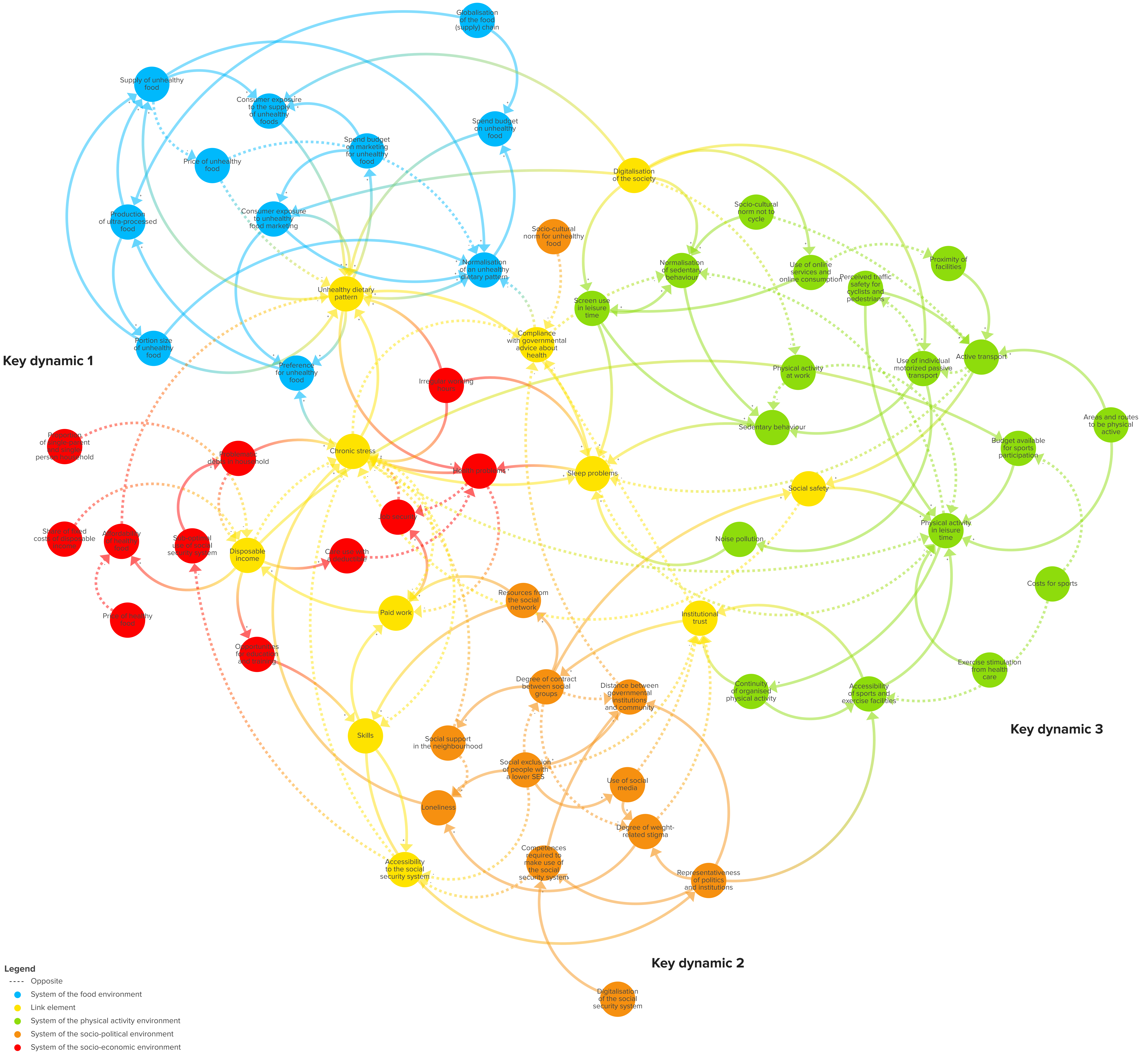

Key dynamic 1
Key dynamic 2
Key dynamic 3
Globalisation of the food (supply) chain
Supply of unhealthy food
Consumer exposure to the supply of unhealthy foods
Spend budget on unhealthy food
Spend budget on marketing for unhealthy food
Price of unhealthy food
Production of ultra-processed food
Consumer exposure to unhealthy food marketing
Normalisation of an unhealthy dietary pattern
Unhealthy dietary pattern
Portion size of unhealthy food
Preference for unhealthy food
Digitalisation of the society
Socio-cultural norm for unhealthy food
Compliance with governmental advice about health
Socio-cultural norm not to cycle
Normalisation of sedentary behaviour
Use of online services and online consumption
Proximity of facilities
Perceived traffic safety for cyclists and pedestrians
Screen use in leisure time
Active transport
Physical activity at work
Use of individual motorized passive transport
Sedentary behaviour
Areas and routes to be physical active
Budget available for sports participation
Irregular working hours
Proportion of single-parent and single-person household
Problematic debts in household
Chronic stress
Health problems
Sleep problems
Social safety
Job security
Share of fixed costs of disposable income
Affordability of healthy food
Sub-optimal use of social security system
Disposable income
Care use with a deductible
Noise pollution
Physical activity in leisure time
Costs for sports
Price of healthy food
Paid work
Resources from the social network
Institutional trust
Opportunities for education and training
Degree of contract between social groups
Distance between governmental institutions and community
Continuity of organised physical activity
Accessibility of sports and exercise facilities
Exercise stimulation from health care
Skills
Social support in the neighbourhood
Social exclusion of people with a lower SES
Use of social media
Loneliness
Degree of weight-related stigma
Competences required to make use of the social security system
Accessibility to the social security system
Representativeness of politics and institutions
Digitalisation of the social security system
Legend
Opposite
System of the food environment
Link element
System of the physical activity environment
System of the socio-political environment
System of the socio-economic environment
