# Supplementary material for: Understanding the dynamics driving obesity in socioeconomically deprived urban neighbourhoods: an expert-based systems map
Source: BMC Med. 2025 Jan 7;23:2. doi: 10.1186/s12916-024-03798-x (PMC11705861; doi:10.1186/s12916-024-03798-x)
Supplement: Supplementary file 4 — Additional file 4: Feedback loops and mechanisms. [file 12916_2024_3798_MOESM4_ESM.pdf]

Additional file 4: Feedback loops and mechanisms per subsystem

<table>
<tr><th>Feedback loops food environment</th><th>Mechanisms</th></tr>
<tr><td>R1 Supply of unhealthy food → relative price of unhealthy foods → unhealthy dietary pattern → supply of unhealthy food</td><td rowspan="4">The availability of unhealthy products has increased in deprived neighbourhoods in Dutch cities. The price of unhealthy food has remained relatively low. The combination of increased availability, low prices, and heightened exposure to these products has led to the normalization of an unhealthy dietary pattern in these neighbourhoods. This normalization, in turn, results in more people adopting an unhealthy diet. Consequently, a higher number of people with an unhealthy diet in the neighbourhood leads to an increased demand for unhealthy food, which, in response, leads to a further increase in the supply of these products.</td></tr>
<tr><td>R2 Supply of unhealthy food → consumer exposure to the supply of unhealthy food → unhealthy dietary pattern → supply of unhealthy food</td></tr>
<tr><td>R3 Supply of unhealthy food → normalisation of an unhealthy dietary pattern → spend budget on unhealthy food → unhealthy dietary pattern → supply of unhealthy food</td></tr>
<tr><td>R4 Supply of unhealthy food → relative price of unhealthy foods → normalization of an unhealthy dietary pattern → spend budget on unhealthy food → unhealthy dietary pattern → supply of unhealthy food</td></tr>
<tr><td>R5 Supply of unhealthy food → normalisation of an unhealthy dietary pattern → spend budget on unhealthy food → unhealthy dietary pattern → preference for unhealthy food → production of ultra-processed food → supply of unhealthy food</td><td rowspan="2">Due to an increased demand for unhealthy food, driven in part by a shift in preferences towards unhealthy options, the production of ultra-processed food has risen. Consequently, this increase in production further expands the supply of and exposure to unhealthy food choices.</td></tr>
<tr><td>R6 Supply of unhealthy food → consumer exposure to the supply of unhealthy food → unhealthy dietary pattern → preference for unhealthy food → production of ultra-processed food → supply of unhealthy food</td></tr>
<tr><td>R7 Normalisation of an unhealthy dietary pattern → spend budget on unhealthy food → unhealthy dietary pattern → spend budget on the marketing for unhealthy food → consumer exposure to unhealthy food marketing → normalization of an unhealthy dietary pattern</td><td rowspan="2">The increased normalization of an unhealthy dietary pattern reinforces its adoption. As the demand for unhealthy food rises, the marketing budget for such products increases, leading to a higher exposure to marketing efforts (both online and offline). This increased marketing of unhealthy food further strengthens the unhealthy food preference and availability and further the normalization of unhealthy eating patterns in these neighbourhoods.</td></tr>
<tr><td>R8 Spend budget on marketing for unhealthy food → consumer exposure to unhealthy food marketing → preference for unhealthy food → production of ultra-processed food → supply of unhealthy food → normalization of unhealthy dietary patterns → spend budget on unhealthy food → unhealthy dietary pattern → spend budget on marketing for unhealthy food</td></tr>
</table>

<table>
<tr><td>R9 Unhealthy dietary pattern → preference for unhealthy food → production of ultra-processed food → portion size of unhealthy food → unhealthy dietary pattern</td><td rowspan="2">The increased production of ultra-processed food leads to larger portions of unhealthy products, making dietary patterns unhealthier and further normalizing this unhealthy eating behaviour. This, in turn, influences the preference for such products, leading to even more production of ultra-processed food. This cycle perpetuates the availability and consumption of unhealthy products, contributing to the increase of unhealthy dietary patterns in the neighbourhood.</td></tr>
<tr><td>R10 Production of ultra-processed food → portion size of unhealthy food → normalisation of an unhealthy dietary pattern → spend budget on unhealthy food → unhealthy dietary pattern → preference for unhealthy food</td></tr>
<tr><td>R11 Preference for unhealthy food → production of ultra-processed food → supply of unhealthy food → relative price of unhealthy foods à unhealthy dietary pattern → preference for unhealthy food</td><td>As the preference shifts towards more unhealthy products, driven by increased marketing and the availability of ultra-processed food, the production and supply of unhealthy products also increase. This results in keeping the prices of these products low due to the demand-supply mechanism.</td></tr>
</table>

**Feedback loops physical activity environment**	**Mechanisms**
R1 Sedentary behaviour → sleep problems → physical activity in leisure time → normalisation of sedentary behaviour → sedentary behaviour	An increase in sedentary behaviour leads to more sleep problems and a decrease in physical activity. When a low number of people engage in physical activity in a neighbourhood, and leisure screen time rises, it fosters a process of normalizing sedentary behaviour within the community. This normalization can further reinforce sedentary behaviour.
R2 Use of individual motorized passive transport → perceived traffic safety for cyclists and pedestrians → active transport → use of individual motorized passive transport	An increase in the use of individual motorized passive transport (such as cars, motorcycles, scooters) results in higher traffic volume and a decrease in perceived safety for cyclists and pedestrians. As a consequence of reduced perceived safety, active transport in these areas declines even further and individual motorized passive transport becomes even more popular.
R3 Accessibility of sports and exercise facilities → physical activity in leisure time → continuity of organised physical activity → accessibility of sports and exercise facilities	The available sports and exercise facilities do not always align with the residents' needs, leading to reduced physical activity levels. This decreases the demand for such facilities, resulting in a decline in the continuity and accessibility of the offerings. Additionally, the pressure on outdoor space may lead to many

	sports facilities moving to the outskirts of the city, making them less accessible to the population.

**Feedback loops socioeconomic environment**	**Mechanisms**
R1 Accessibility to the social security system → chronic stress → skills → accessibility to the social security system	The social security system has become less accessible due to increasing complexity, especially with the digital aspects. As a result, stress levels have risen, and more people are making suboptimal use of the social security system. This suboptimal usage leads to a discrepancy between the amount of benefits and allowances paid out and the actual entitled amount, resulting in individuals having to repay excess amounts, increasing the risk of problematic debts.<br><br>The rise in debts contributes to a further increase in chronic stress. Moreover, accumulating debts lead to a decrease in disposable income, which, in turn, can cause additional stress. Chronic stress can also hinder individuals from focusing on and developing essential skills, including digital and financial literacy, while reduced disposable income may limit opportunities for further education.<br><br>These combined factors exacerbate the already complex social security system, making it even less accessible for those in need
R2 Accessibility to the social security system → sub-optimal use of the social security system → problematic debts in household → chronic stress → skills → accessibility to the social security system	
R3 Accessibility to the social security system → sub-optimal use of the social security system → problematic debts in household → disposable income → chronic stress → skills → accessibility to the social security system → sub-optimal use of the social security system	
R4 Accessibility to the social security system → sub-optimal use of the social security system → problematic debts in household → disposable income → opportunities for education and training → skills → accessibility to the social security system → sub-optimal use of the social security system	
R12 Accessibility to the social security system → chronic stress → paid work → disposable income → opportunities for education and training → skills → accessibility to the social security system	
R5 Disposable income → opportunities for education and training → skills → paid work → disposable income	As the proportion of fixed expenses in disposable income rises and the number of single-parent households and individuals living alone increases in deprived neighbourhoods in

<table>
<tr><td></td><td rowspan="2">Dutch cities, the disposable income decreases. This reduction leads to a decline in opportunities for education and an increase in chronic stress. Insufficient basic skills may affect the ability to secure a well-paying job, thereby perpetuating the cycle of low disposable income.</td></tr>
<tr><td>R6 Disposable income → chronic stress → skills → paid work → disposable income</td></tr>
<tr><td>R7 Disposable income → affordability of healthy food → unhealthy dietary pattern → health problems → paid work → disposable income → affordability of healthy food</td><td rowspan="3">A low disposable income and the high cost of healthy food encourage an unhealthy dietary pattern, leading to an increase in health issues, both physical and mental. These health problems, in turn, reduce job security and escalate chronic stress. Individuals experiencing high levels of chronic stress are more likely to be unemployed.
Furthermore, an increase in chronic stress contributes to sleep problems, exacerbating health problems. The reduced likelihood and extent of paid employment result in a lower disposable income, perpetuating unhealthy eating patterns and chronic stress even further.</td></tr>
<tr><td>R8 Chronic stress → sleep problems → health problems → job security → chronic stress</td></tr>
<tr><td>R14 Health problems → job security → chronic stress → paid work → disposable income → affordability of healthy food → unhealthy dietary pattern → health problems</td></tr>
<tr><td>R10 Disposable income → care use with a deductible → health problems → chronic stress → skills → paid work → disposable income</td><td rowspan="3">A lower disposable income results in reduced access to healthcare service that bring extra costs in the form of excess, costs for care, transport costs or lost income. Avoiding necessary healthcare due to financial constraints leads to an increase in health problems. As physical or mental health issues worsen, they can lead to physical and mental limitations that reduce the chances of finding paid work and, consequently, lower the disposable income even further. The decrease in disposable income and the increase in chronic stress leave less mental capacity and attention for developing or strengthening basic skills. This situation also limits opportunities for further education, leading to fewer prospects for paid work and a higher disposable income.</td></tr>
<tr><td>R15 Disposable income → care use with a deductible → health problems → paid work → disposable income → opportunities for education and training → skills → paid work → disposable income</td></tr>
<tr><td>R16 Disposable income → care use with a deductible → health problems → chronic stress → skills → paid work → disposable income</td></tr>
</table>

**Feedback loops socio-political environment**	**Mechanisms**
R2 Distance between governmental institutions and residents → social exclusion of people with a lower SES → degree of contact between social groups → distance between governmental institutions and residents	Due to a decrease in the representation of residents from deprived neighbourhoods in politics and institutions, and the increasing digitalization that requires more skills to access government systems, the gap between the government, institutions, and the community in the neighbourhood widens.<br><br>This increased distance results in a rise in social exclusion for residents living in deprived neighbourhoods and reinforces a decline in their trust in societal and political institutions, organizations, and establishments.
R3 Distance between governmental institutions and residents → social exclusion of people with a lower SES → institutional trust → degree of contact between social groups → distance between governmental institutions and residents	
R1 Degree of contact between social groups → degree of weight-related stigma → institutional trust → degree of contact between social groups	Through the use of social media, institutional trust can be further reinforced, but at the same time, weight-related stigma can also be exacerbated. Both of these effects can contribute to a further decrease in contact between social groups, leading to an increase in the distance between the government, institutions, and the residents in the neighbourhood.
R4 Institutional trust → degree of contact between social groups → distance between governmental institutions and residents → social exclusion of people with a lower SES → use of social media → institutional trust	
R5 Degree of contact between social groups → distance between governmental institutions and residents → social exclusion of people with a lower SES → use of social media → degree of weight-related stigma → institutional trust → degree of contact between social groups	
R6 Representativeness of politics and institutions → degree of weight-related stigma → institutional trust → degree of contact between social groups → resources from the social network → skills → representativeness of politics and institutions	Due to the decrease in representation of residents from deprived areas in politics and institutions, policies may not align with the needs of these groups. This mismatch results in increased weight-related stigmatization, higher skill requirements for using government systems, and a growing gap between the government and the community in the neighbourhood.<br><br>These processes contribute to the exclusion of lower socioeconomic groups and foster
R7 Representativeness of politics and institutions → distance between governmental institutions and residents → social exclusion of people with a lower SES → degree of contact between social groups → resources from the	

<table>
<tr><td>social network → skills → representativeness of politics and institutions</td><td rowspan="4">institutional distrust, particularly through social media usage. As a consequence, contact between different social groups decreases, reinforcing the distance between the government and the community.

With fewer social resources available due to decreased contact between social groups, the development of skills also declines. As a result, opportunities to attain higher positions within the government and organizations decrease, further reinforcing the decrease in representation of communities.</td></tr>
<tr><td>R8 Representativeness of politics and institutions → competences required to make use of the social security system → distance between governmental institutions and residents → social exclusion of people with a lower SES → degree of contact between social groups → resources from the social network → skills → representativeness of politics and institutions</td></tr>
<tr><td>R9 Representativeness of politics and institutions → competences required to make use of the social security system → distance between governmental institutions and residents → social exclusion of people with a lower SES → use of social media → degree of weight-related stigma → institutional trust → degree of contact between social groups → resources from the social network → skills → representativeness of politics and institutions</td></tr>
<tr><td>R10 Representativeness of politics and institutions → competences required to make use of the social security system → distance between governmental institutions and residents → social exclusion of people with a lower SES → institutional trust → degree of contact between social groups → resources from the social network → skills → representativeness of politics and institutions</td></tr>
</table>
