# Supplementary material for: Understanding the dynamics driving obesity in socioeconomically deprived urban neighbourhoods: an expert-based systems map
Source: BMC Med. 2025 Jan 7;23:2. doi: 10.1186/s12916-024-03798-x (PMC11705861; doi:10.1186/s12916-024-03798-x)
Supplement: Supplementary file 5 — Additional file 5: Definitions of factors. [file 12916_2024_3798_MOESM5_ESM.pdf]

Additional file 5: Definitions of factors included in the CLD

**Factor**	**Definition**
Globalisation of the food (supply) chain	Domination of the food chain by a few multinationals
Production of ultra-processed foods	Production of ready-to-eat food, often with five or more ingredients and with additives such as synthetic dyes, flavour enhancers and emulsifiers (NOVA classification). Examples are chips, cookies, ice cream, burgers, soft drinks and many ready meals.
Consumer exposure to unhealthy food marketing	Degree of consumer exposure to the marketing of unhealthy foods
Price of unhealthy food	The cost of unhealthy food in supermarkets and other locations where unhealthy food is for sale
Supply of unhealthy food	Different locations/contexts where unhealthy food is offered and the amount of unhealthy food offered at these locations
Compliance with governmental advice about health	Proportion of residents who adhere to the guidance and recommendations provided by governmental bodies and associated institutions regarding maintaining and improving their health
Portion size of unhealthy food	The amount of food or drink per portion of an unhealthy product
Preference for unhealthy food	Proportion of residents who have acquired or developed a preference for unhealthy food
Spend budget on marketing for unhealthy food	Spend money on marketing for unhealthy food in the neighbourhood
Spend budget on unhealthy food	Proportion of disposable income spend on unhealthy food per household
Unhealthy dietary pattern	Proportion of residents with an unhealthy diet. A diet that consists of eating (too) little fruit and vegetables or generally eating a lot or eating or drinking unhealthy foods. Unhealthy foods are sweetened drinks, products with too much salt, sugar or saturated fat and fast food (based on RIVM, 2021)

Normalisation of an unhealthy dietary pattern	An unhealthy dietary pattern becomes normal and seen as the norm
Digitalisation of the society	The ongoing integration of digital technologies and digitised data across society
Consumer exposure to the supply of unhealthy food	Degree of exposure of consumers to unhealthy food (vs. healthy food) in the neighbourhood, including through the positioning of unhealthy food in the supermarket, frames to attract attention, but also fast food chains, counters on stations, social media, etc.
Chronic stress	Proportion of residents with chronic stress, defined as physical or emotional exhaustion due to prolonged exposure to stressors or circumstances beyond your control (based on Juster, 2010; Hosper & van Loenen, 2021)
Areas and routes to be physical active	Availability of properly maintained exercise gardens, parks, allotments, walking paths, rollerblading tracks, cycle paths, and routes conducive to physical activities and daily exercise
Social safety	Both feelings of insecurity and actual crime and nuisance in the neighbourhood. Nuisance can be physical (graffiti) or social (loitering youth).
Noise pollution	Presence of intrusive and disruptive sounds within the neighbourhood, originating from vehicular traffic, neighbouring activities, construction projects, or pedestrian commotion, causing discomfort and annoyance
Use of online services and online consumption	Proportion of residents ordering online and using online services and products
Active transport	Proportion of residents traveling on foot, by bicycle or e-bike

Normalisation of sedentary behaviour	Sedentary behaviour becomes normal and seen as the norm
Accessibility of sports and exercise facilities	The extent to which sports facilities offered by sports associations or gyms are accessible, affordable, conveniently located, and usable for residents
Exercise stimulation from health care	Degree of commitment of healthcare professionals to get residents to exercise more and better
Sleep problems	Proportion of residents encountering sleep deprivation, insomnia and poor quality of sleep
Proximity of facilities	Proximity to necessary facilities in the neighbourhood (schools, libraries, shops, work, care centres, hospitals)
Physical activity in leisure time	Engagement of residents in various forms of movement-based endeavours during their free time, including activities like walking, cycling, running, visits to gyms or sports clubs for enjoyment and health benefits.
Continuity of organised physical activity	Continuation of current organised initiatives and programs in the neighbourhood, aimed at sports and exercise instead of constantly setting up and breaking down new short-term initiatives
Costs for sports	The costs for a sport membership or sports club subscription, costs to participate in sports activities, costs for sports equipment
Sedentary behaviour	Engagement of residents in waking behaviour characterized by a low energy expenditure while in a sitting, reclining or lying posture. Common sedentary behaviours include watching TV, playing video games, using computers, driving, and reading (based on Tremblay et al., 2017)
Socio-cultural norm not to cycle	The extent to which the use of e.g. scooter, car, metro and tram is the unwritten, accepted standard in a community instead of using the bicycle
Screen use in leisure time	The amount of leisure time residents spent interacting with media screens including television, tablets, laptops, gaming and smartphones
Physical activity at work	Residents' engagement in moderate to intense physical activity during their work hours. This includes measuring factors like the number of steps taken, as well as involvement in physically demanding occupations such as hairdressing or masonry.

Perceived traffic safety for cyclists and pedestrians	The degree of perceived safety in road traffic for cyclists and pedestrians in the neighbourhood
Use of individual motorized passive transport	Use of cars, scooters, motorcycles and mobility scooters in the neighbourhood
Degree of weight-related stigma	Negative attitudes and beliefs in society that manifest themselves in the form of prejudices, stereotypes and exclusion towards adults because they are overweight (based on Noordam, 2016).
Use of social media	Use of online platforms such as Twitter, Facebook and Instagram by residents
Paid work	Proportion of residents who receive a financial benefit/salary for the work they do on the basis of an employment contract
Social support in the neighbourhood	This includes informational, instrumental and emotional social support from other residents of the neighbourhood or professionals in the neighbourhood (e.g. community centre) (based on House, 1981; Grassley, 2010).
Digitalisation of the social security system	Digitisation of the facilities, information and services of the social security system (social services and social insurance)
Competences required to make use of the social security system	Competencies that are required in our society, including financial and digital competencies, to make good use of the (digital) social security system, such as applying for benefits, using DigiD (online identification for websites and services of the Dutch government), online tax returns and care-related matters
Distance between governmental institutions and community	A gap in preferences, perspectives and practices between those who work for and within governmental institutions and communities
Resources from the social network	Residents' social contacts in the neighbourhood with knowledge and skills that can help achieve intended goals, such as success in the labour or housing market or influence on (political) decision-making

Degree of contact between social groups	Degree of (informal) contact between various societal groups both within and between neighbourhoods (e.g., between younger and older people, across educational or income groups, between people with different religious backgrounds, or between people with and without a migration background)
Representativeness of politics and institutions	Percentage of representatives in (local and national) politics with a lower educational background
Accessibility to the social security system	The extent to which the facilities, information and services of the social security system (social services and social insurance) are accessible, understandable and usable for residents
Socio-cultural norm for unhealthy food	The extent to which the consumption of unhealthy food is the unwritten, accepted standard in a community instead of the consumption of healthy food
Loneliness	Proportion of residents who find themselves in a situation characterized by an unpleasant or unacceptable absence of (quality of) relationships with others (based on de Jong-Gierveld, 1998)
Institutional trust	Trust of residents in social and political institutions and organizations (based on CBS, 2022)
Skills	Residents possessing the essential skills to actively participate in society. This concerns basic skills in the areas of language (reading, writing and oral language skills), arithmetic and digital skills (based on Inspectorate of Education 2021; Movisie, 2022)
Social exclusion of people with a lower socioeconomic status	Social exclusion is characterized by limited social participation, financial limitations, a fading sense of norms and disadvantage in access to assistance, care institutions and housing (based on Coumans, 2012).
Disposable income	Gross income minus premiums and taxes paid per household living in deprived urban neighbourhoods
Sub-optimal use of social security system	The difference between what the government pays for rent, childcare, healthcare benefits, and child-related expenses, compared to what a household should receive, along with/or any debts caused by the amount and

	volatility of repayments of benefits received and/or overdue healthcare premiums
Proportion of single-parent and single-person household	Proportion of households consisting of one parent with one or more children living at home or person who lives alone and forms a single-person household (based on CBS, 2023)
Problematic debts in household	Proportion of households in the neighbourhood with problematic debts. Both registered debts that are characterised as problematic following a list of criteria compiled by Statistics Netherlands, and debts that cannot be found in a registration, for example debts owed to family members or friends, are considered
Health problems	Proportion of residents with health problems, including disorders, illnesses but also psychological problems such as depression
Job security	The extent to which individuals can expect to obtain and maintain regular, secure and remunerative employment of their working lives, and to which working conditions enable them to achieve an adequate level of economic and social security
Care use with a deductible	Care use that entails extra costs in the form of excess, costs for care, transport costs or lost income
Irregular working hours	Proportion of residents working outside office hours: evening work, night work or weekend work (based on CBS, 2022)
Share of fixed costs of disposable income	Proportion of household disposable income spend on rent/mortgage, gas and electricity, water, local chargers, telephone, TV, internet, insurance, education, childcare and transport (based on Nibud, 2019)
Affordability of healthy food	The extent to which households can purchase healthy food within the context of their available financial resources
Price of healthy food	The cost of healthy food in supermarkets and other locations where healthy food is for sale
Opportunities for education and training	Opportunities for residents to acquire new skills and enhance existing ones through reskilling and upskilling
Budget available for sports participation	Resident's available budget for a sport membership or sports club subscription, participation in sports activities and sports equipment

Definitions based on:

Movisie. (2022). *Basisvaardigheden.* Available from: https://www.movisie.nl/basisvaardigheden

Centraal Bureau voor de Statistiek. (2023). *Onregelmatige werktijden*. Available from: https://www.cbs.nl/nl-nl/nieuws/2009/42/jongeren-werken-vaak-s-avonds-en-in-het-weekend/onregelmatige-werktijden

Centraal Bureau voor de Statistiek. (2023). *Personen in huishoudens naar leeftijd en geslacht*. Available from: https://www.cbs.nl/nl-nl/cijfers/detail/37620?dl=3EC40

Centraal Bureau voor de Statistiek. (2023). *Sociaal en institutioneel vertrouwen.* Available from: https://www.cbs.nl/nl-nl/nieuws/2022/13/vertrouwen-in-tweede-kamer-steeg-in-2020-en-daalde-in-2021/sociaal-en-institutioneel-vertrouwen

Centraal Bureau voor de Statistiek. (2021). *Huishoudens met geregistreerde problematische schulden* 2015-2021. Available from: Schuldenproblematiek in beeld (cbs.nl)

Coumans, M. (2012). Sociale uitsluiting, beschrijvende analyses.

de Jong Gierveld, J. (1998). A review of loneliness: concept and definitions, determinants and consequences. *Reviews in clinical gerontology, 8*(1), 73-80.

Grassley, J. S. (2010). Adolescent mothers' breastfeeding social support needs. Journal of Obstetric, Gynecologic & Neonatal Nursing, 39(6), 713-722.

Hosper, K., Van Loenen, T. (2021). *Leven met ongezonde stress*.

House, J. S. (1983). Work stress and social support. *Addison-Wesley series on occupational stress.*

Juster, R. P., McEwen, B. S., & Lupien, S. J. (2010). Allostatic load biomarkers of chronic stress and impact on health and cognition. Neuroscience & Biobehavioral Reviews, 35(1), 2-16.

Ministerie van Onderwijs, Cultuur en Wetenschap. (2023). *Basisvaardigheden.* Onderwerp | Inspectie van het onderwijs. Available from: https://www.onderwijsinspectie.nl/onderwerpen/basisvaardigheden

Monteiro, C.A., Cannon, G., Lawrence, M., Costa Louzada, M.L. and Pereira Machado, P. 2019. Ultra-processed foods, diet quality, and health using the NOVA classification system. Rome, FAO.

Nibud (2019). *Meer dan helft van budget op aan vaste lasten*. Available from: https://www.nibud.nl/nieuws/nibud-meer-dan-helft-van-budget-op-aan-vaste-lasten/#:~:text=Het%20Nibud%20ziet%20dat%20huishoudens,iets%20meer%20dan%2050%20procent.

Noordam, H., & Halberstadt, J. (2016). *Factsheet Stigmatisering in de zorg voor kinderen met obesitas. Amsterdam: Care for Obesity.*

RIVM (2021). *Impactvolle determinanten: Ongezonde voeding.* Available from: *LR_012065_131709_Factsheet__ongezonde_voeding_V5.pdf (rivm.nl)*

Tremblay, M.S., Aubert, S., Barnes, J.D. et al. Sedentary Behavior Research Network (SBRN) – Terminology Consensus Project process and outcome. *Int J Behav Nutr Phys Act 14, 75* (2017). https://doi.org/10.1186/s12966-017-0525-8
