# Supplementary material for: Understanding the dynamics driving obesity in socioeconomically deprived urban neighbourhoods: an expert-based systems map
Source: BMC Med. 2025 Jan 7;23:2. doi: 10.1186/s12916-024-03798-x (PMC11705861; doi:10.1186/s12916-024-03798-x)
Supplement: Supplementary file 6 — Additional file 6: Key dynamic 1. [file 12916_2024_3798_MOESM6_ESM.pdf]

Key dynamic 1

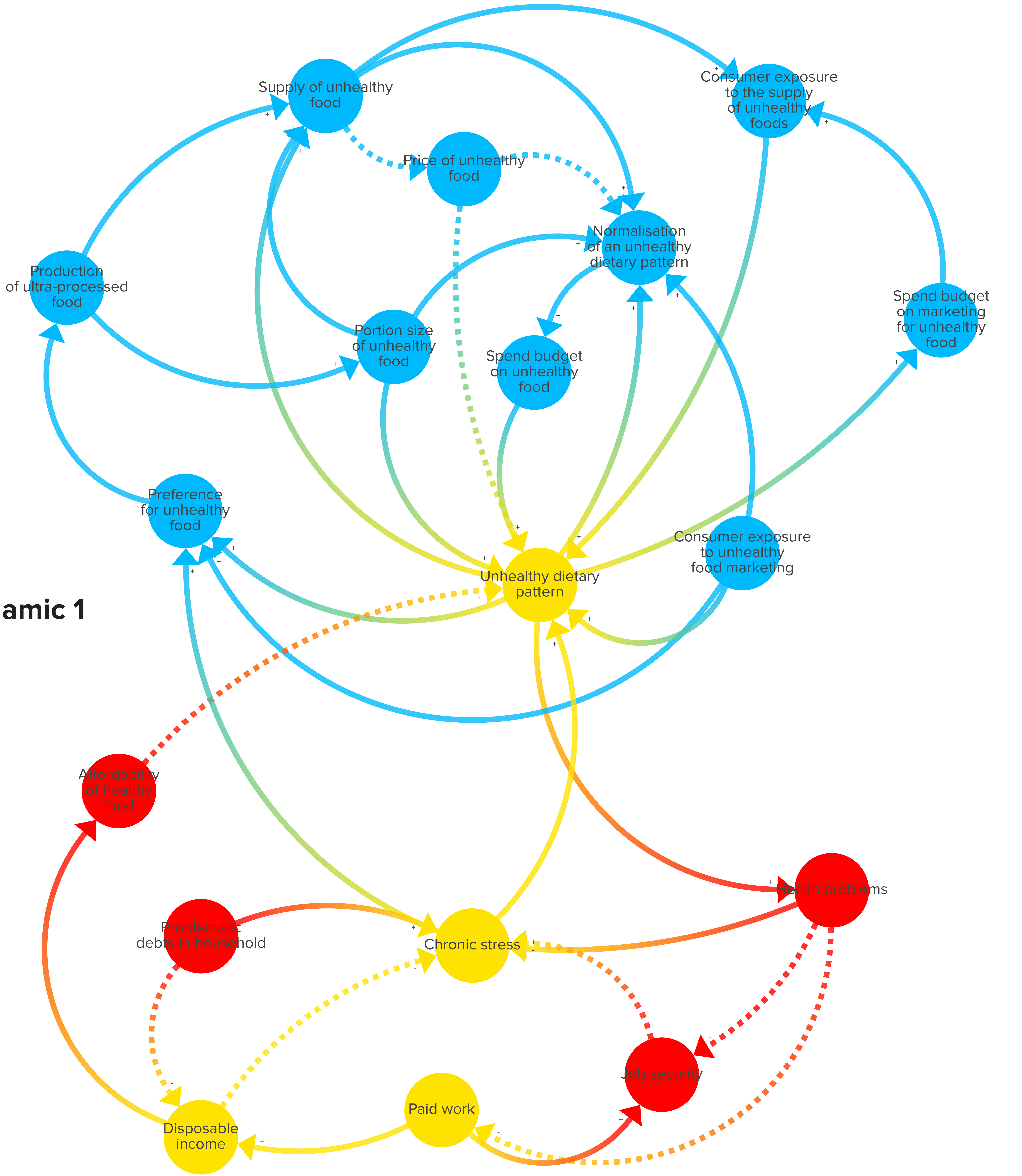

Legend

- - - - Opposite
- System of the food environment
- Link element
- System of the physical activity environment
- System of the socio-political environment
- System of the socio-economic environment
