# Supplementary figures and images for: Understanding the dynamics driving obesity in socioeconomically deprived urban neighbourhoods: an expert-based systems map
Source: BMC Med. 2025 Jan 7;23:2. doi: 10.1186/s12916-024-03798-x (PMC11705861; doi:10.1186/s12916-024-03798-x)

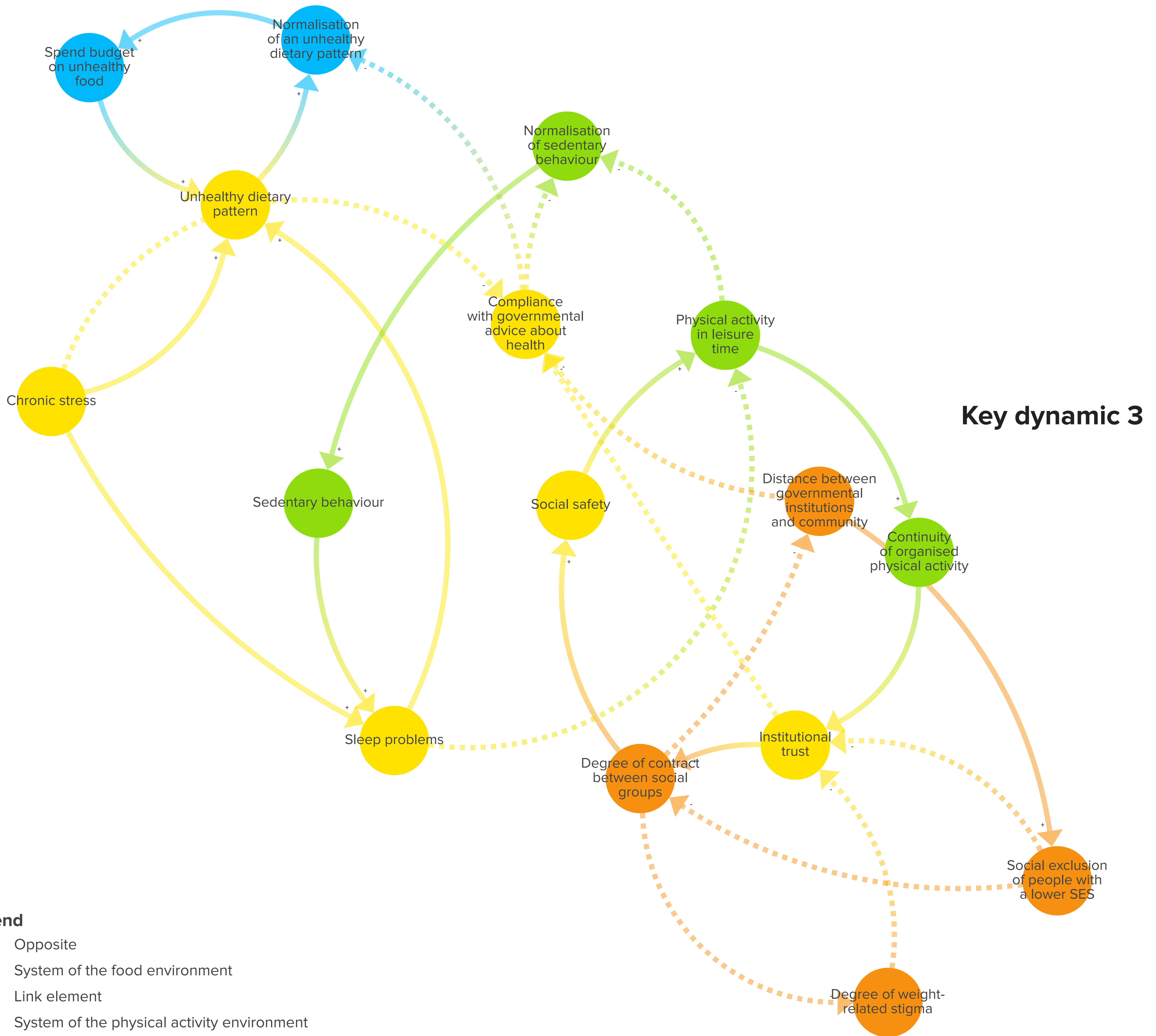

Supplement: Supplementary file 8 — Additional file 8: Key dynamic 3. [file 12916_2024_3798_MOESM8_ESM.pdf]
